# Structural Characterization of Monoclonal Antibodies and Epitope Mapping by FFAP Footprinting

**DOI:** 10.1021/acs.analchem.3c04161

**Published:** 2024-05-03

**Authors:** Lukáš Fojtík, Zuzana Kalaninová, Jan Fiala, Petr Halada, Josef Chmelík, Petr Man, Zdeněk Kukačka, Petr Novák

**Affiliations:** † Institute of Microbiology of the Czech Academy of Sciences, Prague 142 20, Czech Republic; ‡ Faculty of Science, Charles University in Prague, Prague 128 00, Czech Republic

## Abstract

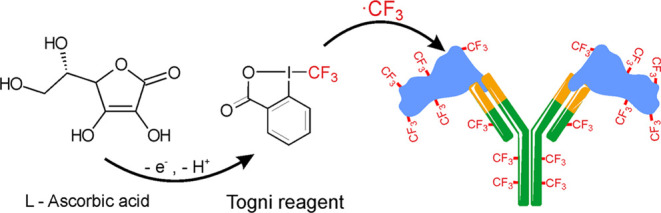

Covalent labeling in combination with mass spectrometry is a powerful approach used in structural biology to study protein structures, interactions, and dynamics. Recently, the toolbox of covalent labeling techniques has been expanded with fast fluoroalkylation of proteins (FFAP). FFAP is a novel radical labeling method that utilizes fluoroalkyl radicals generated from hypervalent Togni reagents for targeting aromatic residues. This report further demonstrates the benefits of FFAP as a new method for structural characterization of therapeutic antibodies and interaction interfaces of antigen–antibody complexes. The results obtained from human trastuzumab and its complex with human epidermal growth factor receptor 2 (HER2) correlate well with previously published structural data and demonstrate the potential of FFAP in structural biology.

Biomolecules, such as monoclonal antibodies (mAb) and other recombinant biopharmaceuticals, have gained significant attention in modern therapy due to their high potential in treating various conditions, including cancer and autoimmune disorders.^1−3^ The first biopharmaceutical approved by the U.S. FDA in 1980 was Humulin, a mAB used for treating diabetes.^4^ The high potential of mAbs as therapeutic agents stems from their ability to have specific interactions with the targeted antigen. Other advantages include fewer side effects, long clearance times, and longer serum half-life.^5,6^ Despite the wide array of clinical benefits that mABs can deliver, their major drawback is often the price. Introduction of biosimilars, biogenerics of original biopharmaceuticals, promotes price competition and leads to better patient access to safe and effective biotherapeutics.^7^ Although their authorization pathway is somewhat simplified, it still needs to conform to a range of stringent requirements. For example, the European Medicines Agency requires biosimilars to exhibit similar quality characteristics, biological activity, safety, and efficacy as their original counterparts. To prove structural identity, it is necessary not only to characterize the primary sequences and post-translational modifications of the expressed proteins but also to demonstrate the proper protein folding and any chemical modifications introduced by production, purification, and long-term storage.

To determine the higher order structure of the antibodies, a broad palette of high-resolution techniques can be considered. This includes nuclear magnetic resonance,^8,9^ X-ray crystallography,^10,11^ and cryo-electron microscopy.^12,13^ Structural mass spectrometry (MS) has been introduced as a new addition to this arsenal of techniques. It can monitor specific conformation changes of antibodies and their dynamics in solution and provide epitope–paratope mapping.^14^ It utilizes several experimental approaches, such as epitope excision,^15^ epitope extraction,^16^ chemical footprinting,^17^ hydrogen–deuterium exchange (HDX),^18,19^ chemical cross-linking,^20^ or fast photochemical oxidation of proteins (FPOP).^21^ The recent discovery of techniques utilizing the radical fluoroalkyl for protein footprinting^22−25^ offers another approach for the structural characterization of therapeutic antibodies and identifying epitope–paratope interacting regions. In this study, we specifically used fast fluoroalkylation of proteins (FFAP) because it is inexpensive and does not require complicated instrumentation.

To monitor the dynamics of trastuzumab, a monoclonal antibody therapeutic molecule marketed under the brand name Herceptin, we selected two batches with different expiration dates. Both trastuzumab batches were modified using the acetic Togni reagent^26,27^ (1-(trifluoromethyl)-1λ5-benzo-[*d*][1,2]iodaoxol-3(1*H*)-one) and acetic imidazole Togni reagent (1-(1,1,2,2-tetrafluoro-2-(1*H*-imidazole-1-yl)ethyl)-1λ3-benzo[*d*][1,2]iodaoxol–3(1*H*)-one) as visualized in Figure 1. In the next experiment, we used FFAP to identify the interaction region of human epidermal growth factor receptor 2 (HER2), the target antigen, with trastuzumab. Standalone molecules of HER2 and trastuzumab and HER2–trastuzumab complex were exposed for 3 s to fluoroalkyl radicals generated by decomposing acetic and acetic imidazole Togni reagents with ascorbate. The results of the experiments are in good agreement with the structural model of HER2–trastuzumab (1N8Z).

**Figure 1:**
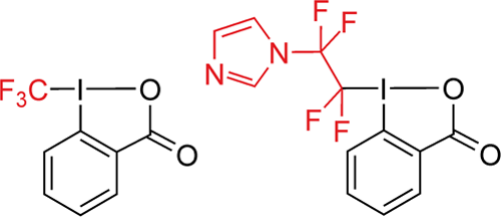
Structures of the cyclic hypervalent iodine-fluoroalkyl reagents used in this study: Acetic Togni reagent (on the left) and acetic imidazole Togni reagent (on the right). The resulting fluoroalkyl radicals are highlighted in red.

## Experimental Section

### Materials

Trastuzumab (Batch N3010H06 with an expiration date of Jan 2022 and Batch H4759H01 with an expiration date of Aug 2021) was purchased from Evidentic GmbH, and HER2 [23–652] was purchased from ProSci. Togni reagents were supplied by CF Plus Chemicals. All other chemicals were purchased from the Sigma-Aldrich Chemical Co. Prior to the FFAP treatment, HER2 was reconstituted according to the manufacturer’s instructions; all protein samples were purified using a size exclusion column (50 mM ammonium bicarbonate pH 7.5, Enrich^TM^ SEC 70, Biorad), and their final concentration was monitored by UV–Vis spectrophotometry (DeNovix DS-11).

### Fast Fluoroalkylation of Proteins

Radical labeling was performed using a capillary flow setup as previously published.^25^ The FFAP flow setup used three syringes pumped with standard syringe pumps. The first syringe was filled with a 1.8 μM solution of the protein (trastuzumab) in 50 mM degassed ammonium bicarbonate buffer (pH 7.5) and 10 mM Togni reagent (the acetic Togni reagent or acetic imidazole Togni reagent); the second syringe contained l-ascorbic acid as a radical inducer with a final concentration of 0.53 mg/mL (2.9 mM), and the third syringe contained l-tryptophan as a quenching solution with a concentration of 10 mg/mL (49 mM). The reaction time was set to 3 and 6 s. The total collection time was 3 min for a 3 s labeling pulse and 6 min for a 6 s labeling pulse, which is equivalent to 10 μg of protein per labeling cycle. For the epitope mapping experiment, the complex of trastuzumab (batch N3010H06 with an expiration date of Jan 2022) with HER2 was analyzed with only a 3 s labeling pulse. The complex of trastuzumab and HER2 was mixed in 1:2 molar ratio. All experiments were performed in triplicates.

### Sample Preparation for MS

Fluoroalkylated samples were precipitated with ice-cold acetone and stored at −80 °C for 1 h. Subsequently, samples were centrifuged for 20 min at 21,000*g*. The resulting pellet was dried at room temperature for 30 min, dissolved in 30 μL of 150 mM 4-ethylmorpholine buffer, containing 6 M guanidine-HCl, 15% AcN, 5 mM Tris(2-carboxyethyl)phosphine (TCEP), and 20 mM chloroacetamide (CAA), pH 8.5, and then reduced and alkylated with TCEP and CAA for 10 min at 70 °C. After alkylation, the samples were diluted three times with liquid chromatography-MS (LC-MS) water and deglycosylated using PNgase F (enzyme/protein ratio 1:20) overnight at 37 °C. The samples were then digested with a combination of trypsin/Lys-C (enzyme/protein ratio: 1:20) at 37 °C for 4 h with subsequent addition of trypsin/LysC proteases. The digestion was stopped after 8 h by adding TFA to give a final concentration of 0.1%.

### LC-MS and LC-MSMS of Fluoroalkylated Samples

Three independently collected FFAP samples were then analyzed by LC-MS and LC-MSMS. In both cases, samples were separated on a reversed-phase column using an Agilent 1290 ultra-performance liquid chromatography (UPLC) under the same previously described conditions.^28^ The peptide mixture was reconstituted with 50 μL of 0.1% formic acid, and 1 μL was injected onto a Luna Omega Polar C18 desalting column (0.3 mm × 30 mm, 5 μm, 100 Å, Phenomenex). After 5 min of desalting at a flow rate of 20 μL/min, the peptides were separated with a Luna Omega Polar C18 analytical column (0.3 mm × 150 mm 3 μm, 100 Å, Phenomenex) at a flow rate of 10 μL/min with a gradient from 2 to 35% of acetonitrile in 40 min. Both columns were heated to 50 °C.

For the LC-MS analysis, the UPLC system was coupled online to a solariX XR FT-ICR mass spectrometer (Bruker Daltonics). The eluted peptides were analyzed with 1 M data points over the range of 250–2500 *m*/*z*. The resulting spectrum was created by averaging 4 subsequent spectra with accumulation of ions in the collision cell for 0.2 s. The mass spectrometer was operated in positive mode, and the analyte at *m*/*z* 922.0098 from the electrospray ionization time-of-flight (ESI-TOF) tuning mix (Agilent Technologies) was used as a lock mass.

For the LC-MSMS analysis, eluted peptides were analyzed on a timsTOF Pro mass spectrometer (Bruker Daltonics) equipped with captive spray. Precursor ions in the *m*/*z* range between 100 and 1700 with charge states ≥2+ and ≤6+ were selected for fragmentation, the target intensity per individual PASEF precursor was set to 1 000, and the intensity threshold was set to 1500. The scan range was set between 0.6 and 1.6 V s/cm^2^ with a ramp time of 100 ms. The number of PASEF MS/MS scans was 10.

### Data Analysis

The raw data were processed by Data Analysis 5.2 software (Bruker Daltonics) and exported in the mascot generic format (mgf). The generated mgf files were searched on the Mascot^29^ (Matrix Science) server with the following setup: protease–trypsin/Lys-C with 3 missed cleavage; fixed modification–carbamidomethylation of cysteines; variable modification–oxidation of methionine, fluoroalkylation of tryptophan, tyrosine, phenylalanine, histidine, and cysteine. The mass error was set at 10 ppm for the precursor ion and at 15 ppm for fragments ions. Mass shifts of 66.9784 and 166.0154 Da were set for modification by trifluoromethyl and tetrafluoroethyl–imidazole radicals, respectively. All fluoroalkylations were checked manually in the spectra.

The extent of modification was quantified from the extracted ion chromatograms that were created for each peptide. The first monoisotope of the most intense charge state at the peak of the chromatographic signal was used for further calculation. The quantification was performed using the identical equation (eq 1) that was used in the FPOP experiment carried out by Michael Gross’s research group.^22^
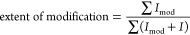
1where *I*_mod_ represents the intensity of the peptide with the modified residue and *I* is the intensity of the peptide with the nonmodified residue. Standard deviation, *P*-value, and *t* test were used as statistical tools.

### Hydrogen–Deuterium Exchange (HDX) Mass Spectrometry

HDX of HER2 and the HER2–trastuzumab complex was initiated by a 10-fold dilution in deuterated buffer (50 mM HEPES pD 7.5, 150 mM NaCl). The final protein concentration of HER2 was 1.7 μM. The molar ratio of HER2 to trastuzumab in the complex was 1:1. Aliquots were collected after 20 s, 1, 5, 20 min, and 2 h, and two time points (5 min and 2 h) were replicated (*n* = 3). The exchange was stopped by adding 0.5 M glycine–HCl buffer pH 2.3, 4 M urea, and 0.25 M TCEP in 1:1 ratio. Samples were incubated for 5 min on ice for disulfide bond reduction and subsequently rapidly frozen in liquid nitrogen. Samples were thawed right before analysis and were immediately injected onto an LC system by a PAL DHR robot operating in manual mode, controlled by Chronos software (Axel Semrau). The LC system consisted of coimmobilized nepenthesin-2/pepsin and PNGaseRc (both bed volume 66 μL, Affipro) connected in series, a trapping column (SecurityGuard ULTRA Cartridge UHPLC Fully Porous Polar C18, 2.1 mm ID, Phenomenex), and an analytical column (Luna Omega Polar C18, 1.6 μm, 100 Å, 1.0 mm × 100 mm, Phenomenex). To minimize back-exchange, the LC system, excluding the protease column, was cooled to 0 °C. Samples were digested, and peptides were desalted with 0.4% formic acid in water driven by the 1260 Infinity II Quarternary pump at 200 μL/min. To elute and separate the desalted peptides, a water–acetonitrile gradient (10–45%; solvent A: 0.1% FA in water, solvent B: 0.1% FA and 2% water in acetonitrile) was used. Gradient elution was done on an Agilent 1290 UPLC system at a flow rate of 40 μL/min. The LC system was coupled to the electrospray ionization source of a timsTOF SCP (Bruker Daltonics) mass spectrometer operating in MS mode with 1 Hz data acquisition and with the tims turned off. The acquired data were peak-picked in DataAnalysis, exported to text files, and processed using DeutEx^30^ software. Data were visualized in MSTools.^31^ For peptide identification, the same LC setup was used but with the mass spectrometer operating in MS/MS mode with PASEF active. The MASCOT (v 2.7, Matrix Science) search engine was used to search LC-MS/MS data against a custom-built database combining a common cRAP.fasta and the sequences of trastuzumab, HER2, pepsin, nepethesin-2, and PNGase Rc. Search parameters were set as follows: precursor tolerance of 10 ppm, fragment ion tolerance of 0.05 Da, variable modifications: Asn deamidation, Cys dehydration, and protein N-term acetylation. Decoy search was enabled, FDR <1%, IonScore >20, and peptide length >5.

The mass spectrometry data have been deposited to the ProteomeXchange Consortium via the PRIDE^32^ partner repository with the data set identifier PXD044326. The program NACCESS 2.1.1.^33^ was used to calculate the solvent-accessible solvent area (SASA) of the HER2–trastuzumab complex (PDB: 1N8Z). The calculation was performed for the CF_3_ radical using the rolling probe algorithm.^33^

## Results and Discussion

FFAP was recently introduced as an inexpensive method that can monitor protein solvent-accessible surface areas and can reflect even minor structural changes in the protein structure. To extend the application of the presented FFAP method to studies of structural characterization of therapeutic antibodies and identification of epitope–paratope interacting regions, we characterized a monoclonal antibody, trastuzumab, alone and in complex with the antigen HER2 whose structure had been partially solved by X-ray crystallography. For the first experiment, we used two batches with different expiration dates: one expiring in August 2021 and the other expiring in January 2022. Both trastuzumab batches were modified using 10 mM acetic Togni reagent or 10 mM acetic imidazole Togni reagent for 3 s. The concentration and labeling time were selected based on previously published data^24,25^ and on the results of preliminary experiments. In these experiments, we observed an increase of doubly modified protein at higher reagent concentrations (Figure S1). In contrast, we did not observe significant differences in modification of the antibody in a bottom-up experiment with a longer labeling pulse (Figure S2).

### FFAP Analysis of the Trastuzumab

Using the acetic Togni reagents, we observed modification of 7 out of 24 residues in the light chain (Figure S3A) and 23 out of 53 residues in the heavy chain of trastuzumab (Figure S4A). This observation is consistent with the localization of these residues on the surface of the molecule and with the solvent accessibility of these regions (Table S1). The same amino acids were labeled using both batches of trastuzumab. However, when we compared the extent of modification, we found elevated levels of modification for the residues F116 and H189 of the light chain and for the residues W47, Y57, W110, H288, and W420 of the heavy chain in the batch of trastuzumab with expiration in January 2022. Differences in fluoroalkylation for these residues were below 2% with the exception of H189 of the light chain, which is the most solvent-accessible aromatic residue in trastuzumab and thus the most sensitive to any change. The differences in modification between the batches are likely due to the reduced stability of the first batch, as the labeling experiment was performed after its expiration date. The modification by imidazole-tetrafluoroethyl radicals formed by the reductive decomposition of the acetic imidazole Togni reagent yielded slightly different results. In total, 3 out of 24 residues in the light chain (Figure S3B) and 10 out of 53 residues in the heavy chain of trastuzumab were fluoroalkylated (Figure S4B). However, all residues modified by the acetic imidazole Togni reagent were also modified by the acetic Togni reagent. Therefore, we hypothesize that the lower number of fluoroalkylated residues formed by the acetic imidazole Togni reagent compared to that of the acetic Togni reagents is caused by the larger size of the imidazole-tetrafluoroethyl radical. Compared to the acetic Togni reagents, we found a higher degree of modification only for W420 of the heavy chain in the batch of trastuzumab with a later expiration date. This example shows that the fluoroalkylation by the larger imidazole-tetrafluoroethyl radical is less sensitive to small conformational changes than is fluoroalkylation by the smaller trifluoromethyl radical.

### FFAP Analysis of the HER2–Trastuzumab Complex Using the Acetic Togni Reagent

**Figure 2:**
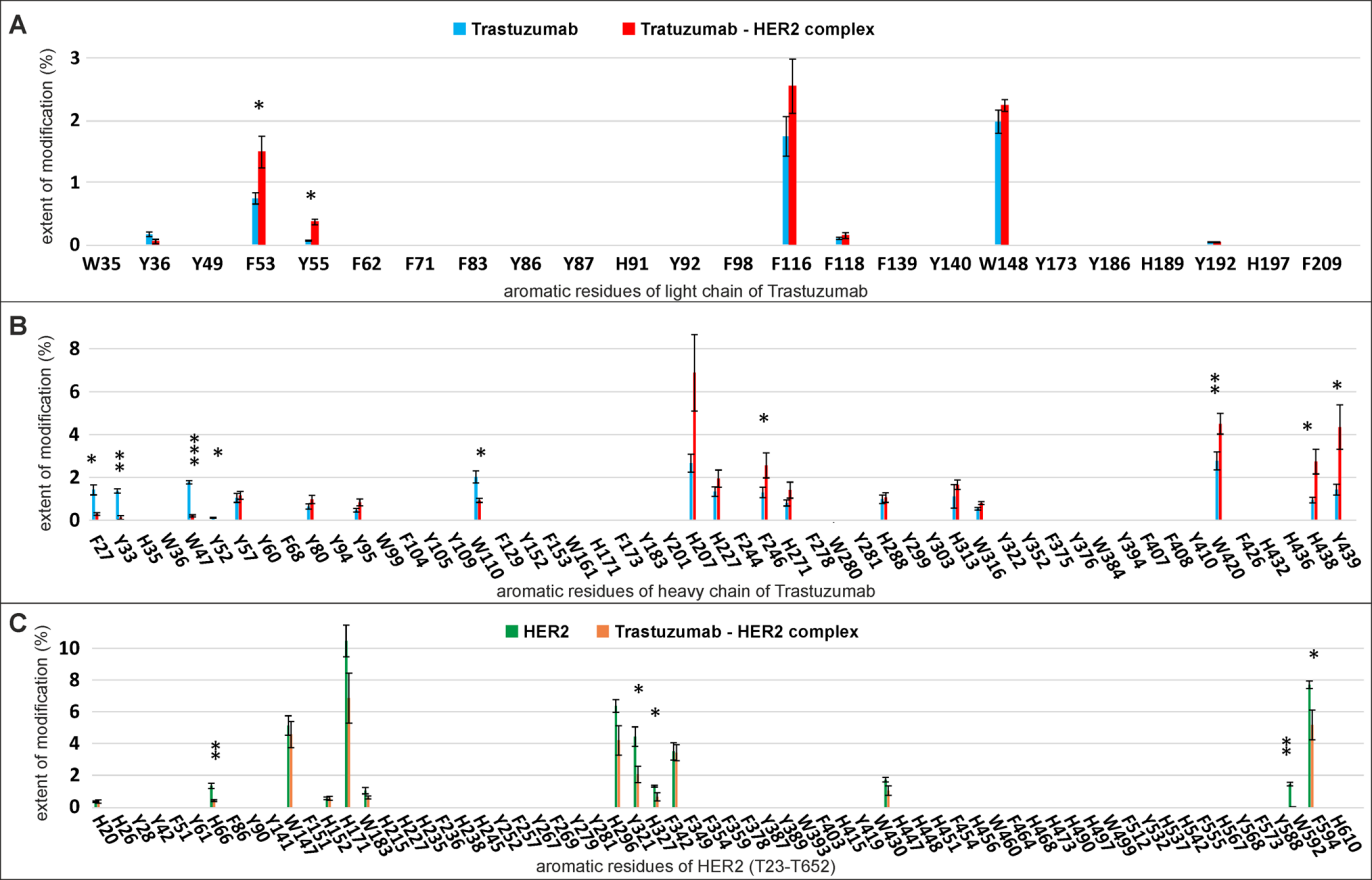
Quantification of the modification for aromatic residues of trastuzumab and in the complex of trastuzumab with HER2. Aromatic residues of (A) light chain, (B) heavy chain of trastuzumab, and (C) HER2 modified by the acetic Togni reagent with a 3 s labeling pulse. The extent of modification of trastuzumab alone (blue bars), the trastuzumab/HER2 complex (red bars), HER2 alone (green bars), and HER2 in complex (orange bars). ***, *P* < 0.005; **, *P* < 0.01; and *, *P* < 0.05.

**Figure 3:**
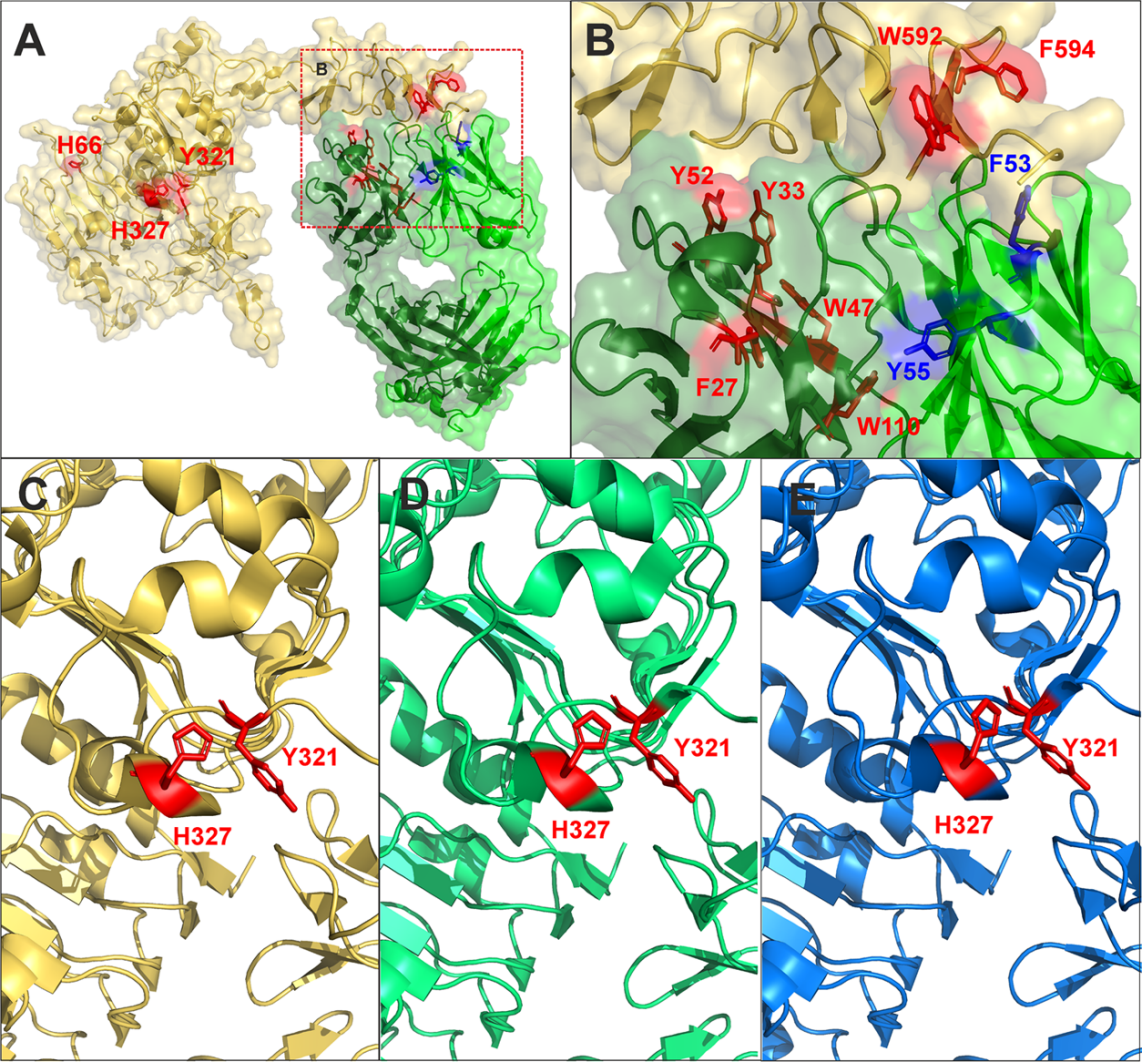
Crystal structures of extracellular domains of HER2 with trastuzumab and pertuzumab. The structure of the extracellular domain of human HER2 with trastuzumab Fab (A). Aromatic residues of trastuzumab (green) and HER2 (yellow) less modified by the acetic Togni reagent in the complex are highlighted in red, while aromatic residues more highly modified in the complex are shown in blue. Zoom-in on the interaction interface of human complex (B). Zoom-in on the extracellular domain of human HER2 (yellow) with trastuzumab (C), extracellular domain of human HER2 (lime green) with pertuzumab (D), and extracellular domain of rat HER2 (blue) (E). Residues Y321 and H327 are highlighted in red.

To investigate the applicability of FFAP for epitope mapping, we characterized the interaction interface of the trastuzumab–HER2 complex. Trastuzumab and HER2 were fluoroalkylated separately and as a complex with both the acetic and acetic imidazole Togni reagents. In the case of the acetic Togni reagent, 7 residues were modified on the light chain, 18 residues were modified on the heavy chain of the antibody, and 13 residues were modified on HER2 (Figure 2). Statistically significant changes between the individual proteins and the complex were observed in 11 residues on the antibody (F53 and Y55 from the light chain and F27, Y33, W47, Y52, W110, F246, W420, H438, and Y439 from the heavy chain) and in 5 residues on HER2 (H66, Y321, H327, W592, and F594). Most of these residues are located on the interaction interface of the complex (Figure 3A), and therefore, there was less modification of these residues in the complex, indicating that their surface is less accessible to the solvent after antigen binding. This correlates well with the data from the crystal structure of the complex. Exceptions are the residues F53 and Y55 from the light chain of the antibody, residues F246, W420, H438, and Y439 from the heavy chain, and H66, Y321, and H327 from HER2. Residues F53 and Y55 from the light chain are in close proximity to the interaction interface of the complex, but they are sticking out of the molecule in the opposite direction toward the interface (Figure 3B). We therefore hypothesize that the side chains of these amino acids were more protected in the structure of the unbound antibody, but conformational changes of this region, induced by the antigen binding, caused their increased exposure. Unfortunately, we do not have any structural information about residues F246, W420, H438, and Y439 from the heavy chain of the antibody, as the structure of the Fc portion of the antibody has not yet been determined. We therefore can only assume that the binding of the antigen to the antibody also caused a structural rearrangement of this region, similar to the residues described above. However, comparing the results of FFAP from the HER2–trastuzumab complex with the crystal structure (Figure 3B) shows a high correlation at the antibody–antigen interaction interface not only in the antigen regions but also on the antibody. This phenomenon indicates that the complementarity determining region (CDR) on the antibody can be determined using the FFAP technique. Residues H66, Y321, and H327, which were less fluoroalkylated in the complex, are part of the HER2 domain responsible for binding to HER3,^34^ which is quite far from the binding site of the antibody. In the crystal structure of the human HER2–trastuzumab complex, Y321 is located on a flexible loop oriented toward H327. In contrast, examination of the HER2 structure of *Rattus norvegicus* (1N8Y) and the structure of the human complex HER2 with pertuzumab (1S78) shows the residue Y321 as part of the β-sheet (Figure 3C–E). This indicates that the decrease in the level of fluoroalkylation of this region could be the result of a more complicated structural rearrangement of the antigen upon antibody binding. Since H327 is part of the β-sheet in all of the structural models described above of HER2, while Y321 is located on a flexible loop only in the human HER2–trastuzumab structure, we can only hypothesize that the reduced modification of residues is caused by their mutual interaction, resulting in either lower accessibility or a change in residue reactivity.

### FFAP Analysis of the HER2–Trastuzumab Complex Using the Acetic Imidazole Togni Reagent

Using the acetic imidazole Togni reagent, 5 residues on the light chain, 19 residues on the heavy chain of the antibody, and 11 residues on the HER2 were modified (Figure S5). Compared to the data from the acetic Togni reagent, the total level of modification of the complex was lower. This observation is consistent with the fact that unmodified residues are less solvent-accessible to the larger imidazole-tetrafluoroethyl radical. Statistically significant changes between individual proteins and proteins in the complex were observed in 10 residues on the antibody (F53 and Y55 from the light chain and F27, Y33, W47, Y52, Y57, Y80, Y109, and W110 from the heavy chain) and in 3 residues on HER2 (W183, W592, and F594). These findings also agree well with the data from the acetic Togni reagent. Compared to the data obtained with the CF_3_ radical, we did not observe statistically significant change in modification of residues F246, W420, H438, and Y439 from the heavy chain of the antibody and residues H66, Y321, and H327 from HER2. F246 was not fluoroalkylated at all because it is not solvent-accessible to the larger imidazole-tetrafluoroethyl radical. For other residues, we noticed differences in modifications between the complex and the individual proteins similar to those in the experiment with the CF_3_ radical, but these differences were not determined to be statistically significant. In the data from the acetic imidazole Togni reagent, we detected changes in the modification of residues Y80, Y109, and W183. Y80 and Y109 are located on the heavy chain of trastuzumab close to the interaction interface, and unlike W183, which is on HER2, it can be assumed that they are eminently affected by antigen binding. In the case of W183 from HER2, the calculated extent of modification is below 1%, and thus, its structure change is minimal. All statistically significant modifications detected in our work were visualized in the previously published crystal structure of the extracellular domain of human HER2 with trastuzumab Fab (Figure S6).

### HDX of the HER2–Trastuzumab Complex

**Figure 4:**
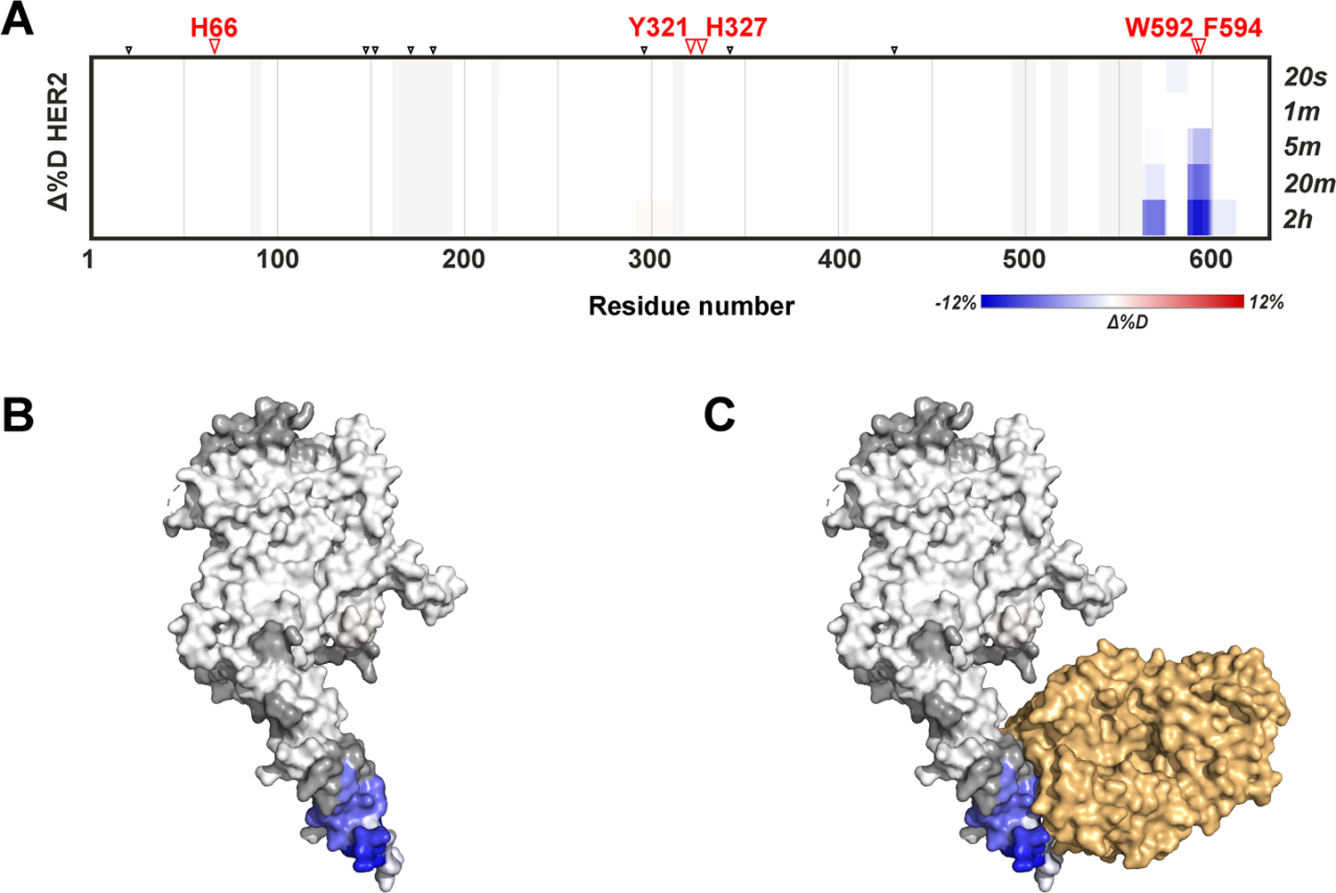
HER2–trastuzumab interaction probed by HDX-MS. Differential heatmap showing differences in deuteration between HER2 alone and in the presence of trastuzumab over the entire HDX kinetics. Blue means protection from deuteration upon trastuzumab binding; whole red stands for higher deuteration (the scale is shown below the heatmap). A clear epitope is visible close to the C-terminus. Residues with significant changes in fluoroalkylation are shown in red above the heatmap. Positions of other reactive residues are highlighted by black arrowheads. Deuteration data from overlapping peptides were recalculated to the shortest nonoverlapping segments as described previously.^37^ Differences in deuteration after 2 h of exchange (indicated on the right side of the heatmap by a black arrowhead) visualized using the same color coding on the structure of human HER2 (B) and human HER2 in complex with the Fab fragment of trastuzumab (C) highlighted in light orange.

To investigate the HER2–trastuzumab interaction at the protein backbone level, HDX-MS measurements of HER2 separately and in complex with trastuzumab were performed. Results presented in the heatmap in Figure 4 demonstrate decreased deuteration of HER2 close to the C-terminus in the presence of trastuzumab. More detailed HDX uptake plots (Figure S7) showed that peptides with the biggest change in deuteration are 587–594 and 590–597, which are in agreement with the X-ray structure of the complex as well as with the decreased fluoroalkylation of residues W592 and F594. Additionally, a smaller change in HER2 protection after trastuzumab binding can also be observed within peptides 565 and 574, corresponding to the modification of F573. Modification of F573 was observed by FFAP within peptide 570–593, that was 3 times fluoroalkylated (W592 doubly and F573 singly) using both the acetic Togni reagent and acetic imidazole Togni reagent. Both peptides had intensities below the quantification limit and were only detected in the case of HER2 study without trastuzumab, which is consistent with solvent inaccessibility of this region in complex (Table S1). Labeling of peptides 570–593 indicates that the fluoroalkylation of W592 to the second degree occurs first, followed by the fluoroalkylation of F573. This finding agrees with the higher solvent accessibility of W592 over that of F573. In contrast, no significant changes in deuteration were observed in the regions containing residues H66, H321, and H327, which may not contradict results of FFAP. Considering that HDX monitors changes in the structure of the protein backbone, while FFAP monitors changes in the structure of side chains, if there is only a small change in the accessibility or reactivity of the side chain that does not affect the peptide backbone, this change will not be reflected in the HDX data but will be reflected in the FFAP data. Therefore, in light of HDX data, we lean toward the theory that the less fluoroalkylation of residues H66, H321, and H327 of HER2 upon trastuzumab binding indicates that the reactivity of aromatic rings on side chains of residues was changed as a result of mutual interaction and that this can be trapped using the FFAP technique. The disadvantage of HDX in the case of the studied complex is the incomplete sequence coverage of the protein (Figure S8). Reduction of HER-2 in the HDX workflow is complicated, and therefore, it was not possible to achieve full coverage with high-intensity signals. In contrast, FFAP labeling allows easier reduction and cleavage of samples, resulting in a higher resolution of the method. An example of this is the region of 152–193, where we observed fluoroalkylation of residues H152, H171, and H183 (Figure 2C), however, which is not covered by HDX. Sequence maps for HER2 and trastuzumab under all studied conditions visualized in MSTools^31^ are provided in the Supporting Information (Figures S9–S14). To improve the sequence coverage of HER2, we utilized online deglycosylation and used quench conditions compatible with PNGase Rc.^35^ They are however milder than required for proper denaturation and reduction of antibodies, and thus, the antibody coverage is compromised, especially in the N-teminal half of the light chain and part of the heavy chain.^36^ Therefore, the antibody deuteration profile was not followed, and the conditions where the antibody is alone were not included.

## Conclusions

FFAP is a novel radical labeling technique based on the covalent modification of aromatic residues by fluoroalkyl radicals. The pioneering work utilizing the FFAP has previously demonstrated its benefits in analyzing solvent-accessible surface areas and protein footprinting. In the current study, we extended the application of FFAP to epitope mapping and to the analysis of a general antigen–antibody complex. The data obtained from human complex trastuzumab–HER2 show a high level of correlation with structural models and demonstrate the usefulness and potential of FFAP in structural biology.

## Supporting Information Available

Comparison of MS spectra for the radical reaction with different labeling times and concentrations of the Togni reagent; quantification plots of the modification for aromatic residues of trastuzumab for both batches studied, and sequence coverages of HER2 and trastuzumab probed by HDX-MS and FFAP (PDF)
