# Supplementary material for: Structural Characterization of Monoclonal Antibodies and Epitope Mapping by FFAP Footprinting
Source: Anal Chem. 2024 May 3;96(19):7386–93. doi: 10.1021/acs.analchem.3c04161 (PMC11099888; doi:10.1021/acs.analchem.3c04161)
Supplement: Supplementary file 1 — ac3c04161_si_001.pdf [file ac3c04161_si_001.pdf]

Supporting Information

Structural Characterisation of Monoclonal Antibodies and Epitope Mapping by FFAP

Lukáš Fojtík[†,‡], Zuzana Kalaninová[†,‡], Jan Fiala[†], Petr Halada[†], Josef Chmelík[†], Petr Man[†], Zdeněk Kukačka[†], and Petr Novák[*,†,‡]

[†]Institute of Microbiology of the Czech Academy of Sciences, Prague, Czech Republic; [‡]Faculty of Science, Charles University in Prague, Prague, Czech Republic

The content in supporting information

1. Figure S1 - Comparison of zoomed MS spectra for all reactions of Trastuzumab with Acetic Togni reagent
2. Figure S2 - Quantification of the modification for aromatic residues of the light chain of Trastuzumab after 3 and 6 s long reaction
3. Figure S3 - Quantification of the modification for aromatic residues of the ligh chain of Trastuzumab
4. Figure S4 - Quantification of the modification for aromatic residues of the heavy chain of Trastuzumab
5. Figure S5 - Quantification of the modification for aromatic residues of Trastuzumab and complex of Trastuzumab with HER2
6. Figure S6 - Crystal structure of extracellular domain of human HER2 with Trastuzumab Fab with highlighted modified residues after reaction with Acetic imidazole Togni reagent
7. Figure S7 - HER2-Trastuzumab interaction probed by HDX-MS in deuterium uptake plots
8. Figure S8 - Sequence coverage of HER2 in HDX-MS
9. Figure S9 - Sequence coverage of HER2 probed by FFAP using the Acetic Togni reagent
10. Figure S10 - Sequence coverage of HER2 probed by FFAP using the Acetic Imidazole Togni reagent
11. Figure S11 - Sequence coverage of Trastuzumab light chain probed by FFAP using the Acetic Togni reagent
12. Figure S12 - Sequence coverage of Trastuzumab heavy chain probed by FFAP using the Acetic Togni reagent
13. Figure S13 - Sequence coverage of Trastuzumab light chain probed by FFAP using the Acetic ImidazoleTogni reagent
14. Figure S14 - Sequence coverage of Trastuzumab heavy chain probed by FFAP using the Acetic Imidazole Togni reagent
15. Table S1 - Table of surface-accessible solvent area for crystal structure of extracellular domain of human HER2 with Trastuzumab Fab

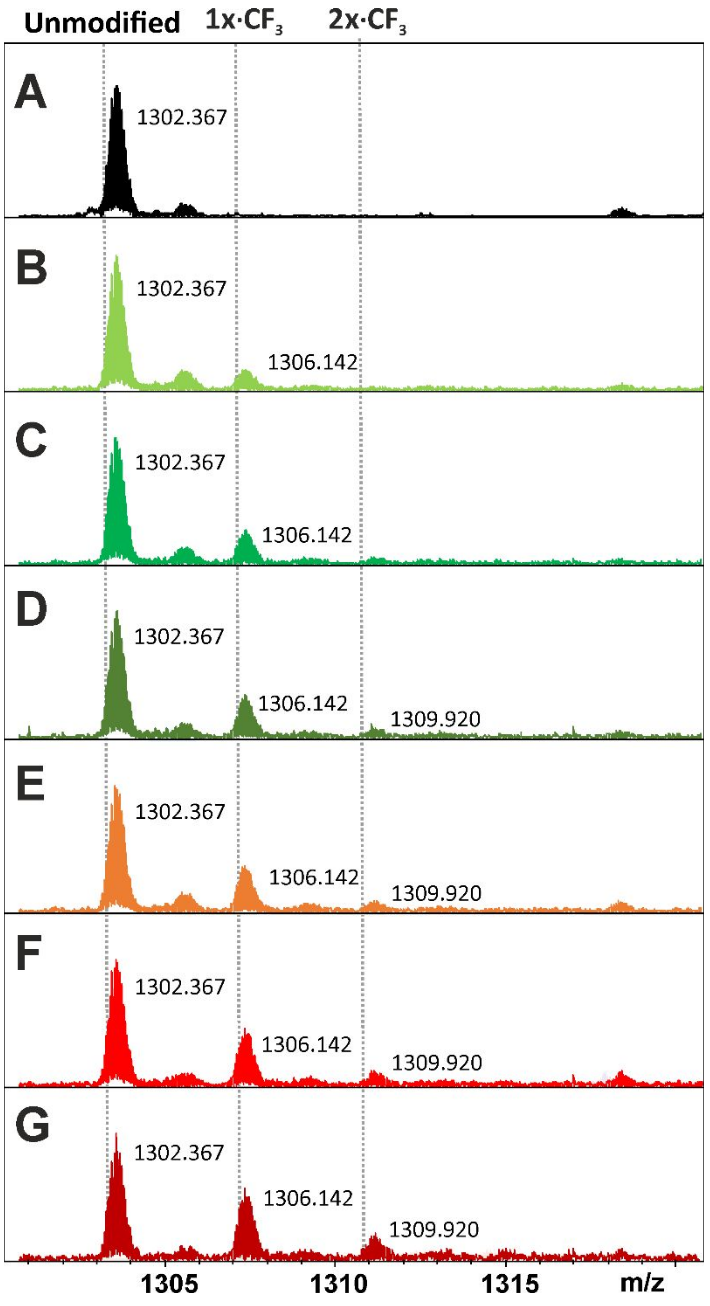

Figure S1: Comparison of MS spectra (zoom on 18+ charge state) for reactions of Trastuzumab light chain with Acetic Togni reagent showing different products of reaction by using different concentration of reagent and different labeling times. Grey lines underline isopic patterns of unmodified, singly and doubly trifluoromethylated light chain of Trastuzumab. MS spectrum of unmodified ligh chain (A), light chain modified by 7.5mM reagent in 3s pulse (B), light chain modified by 10mM reagent in 3s pulse (C), light chain modified by 13mM reagent in 3s pulse (D), light chain modified by 7.5mM reagent in 6s pulse (E) , light chain modified by 10mM reagent in 6s pulse (F), light chain modified by 13mM reagent in 6s pulse (G) .

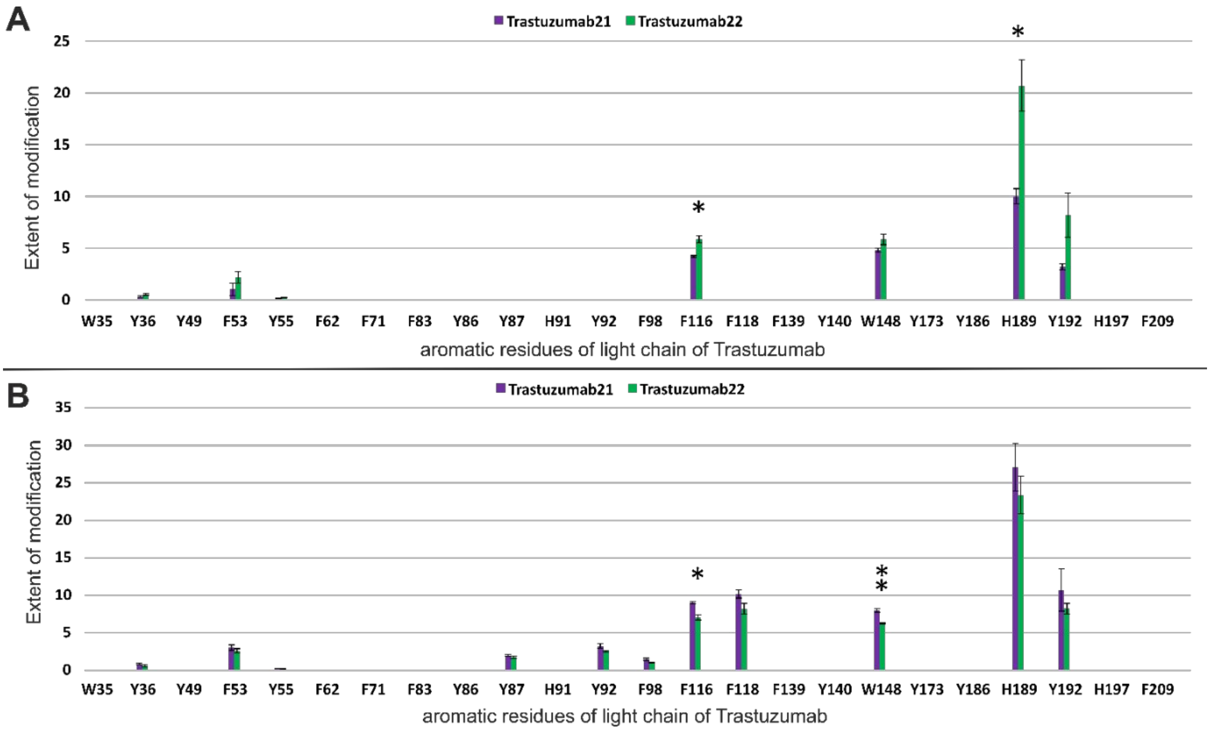

Figure S2 Quantification of the modification for aromatic residues of the light chain of Trastuzumab. Fluoroalkylation by Acetic Togni reagent for 3 s (A) and 6 s (B). Purple bars represent the extent of modification of Trastuzumab expired in August 2021, and green bars of Trastuzumab expired in January 2022. ***, P < 0.005; **, P < 0.01; *, P < 0.05.

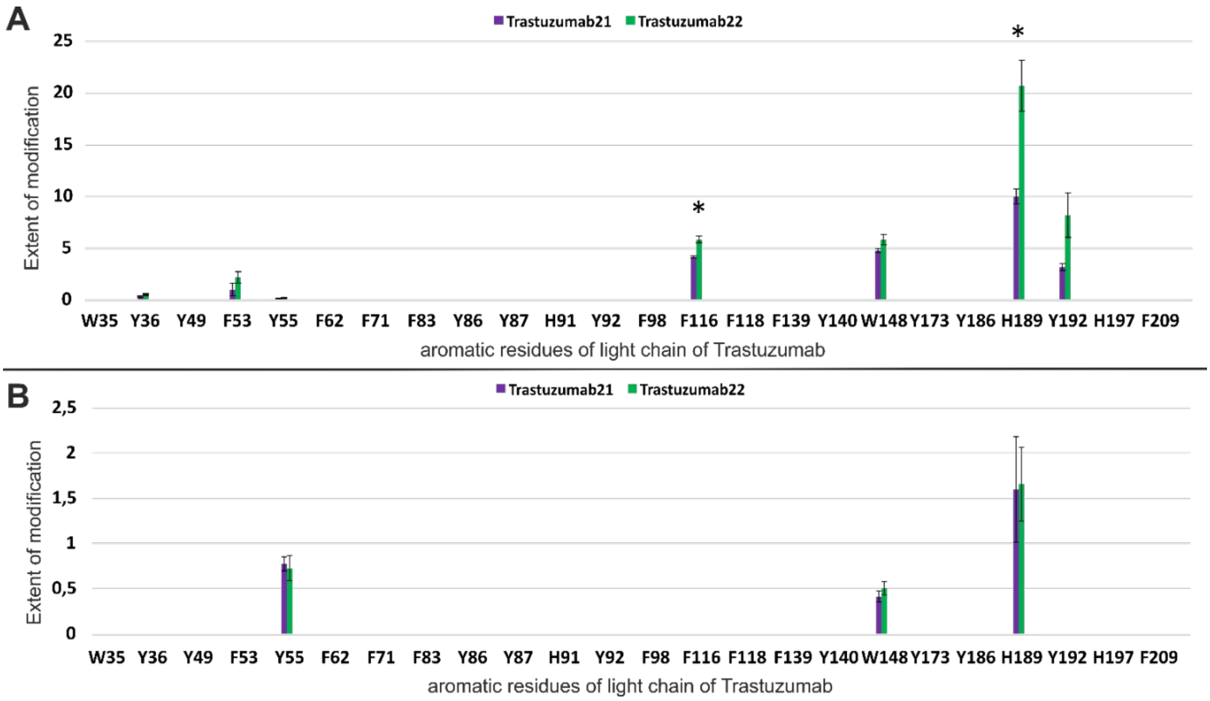

Figure S3 Quantification of the modification for aromatic residues of the light chain of Trastuzumab. (A) Fluoroalkylation by Acetic Togni reagent and (B) by Acetic imidazole Togni reagent. Purple bars represent the extent of modification of Trastuzumab expired in August 2021, and green bars of Trastuzumab expired in January 2022. ***, P < 0.005; **, P < 0.01; *, P < 0.05.

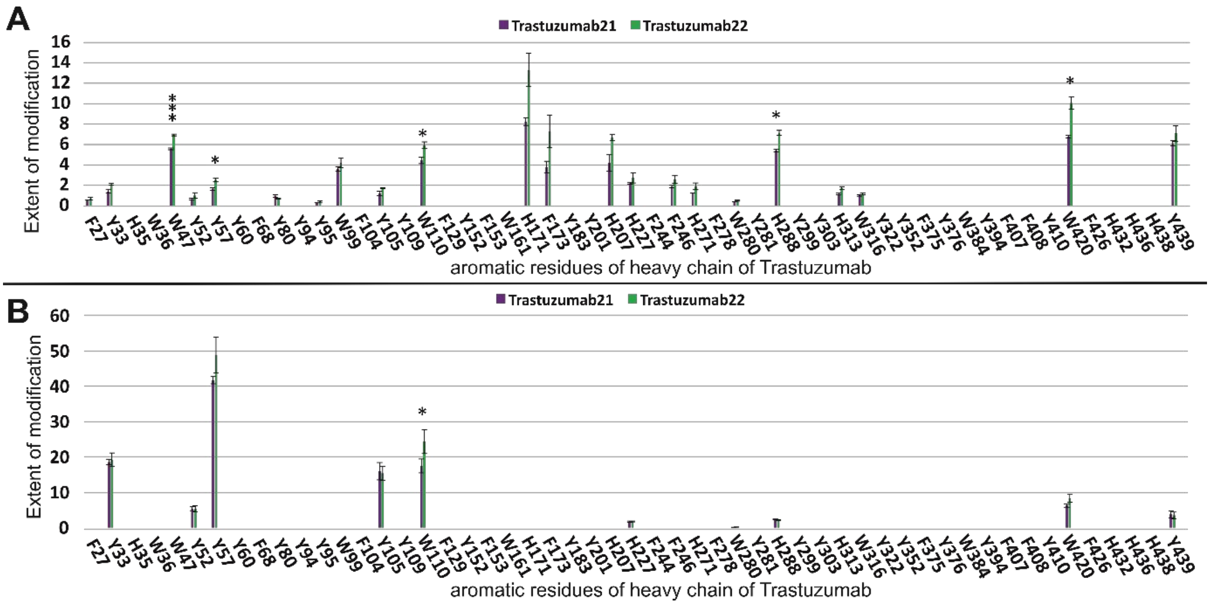

Figure S4. Quantification of the modification for aromatic residues of the heavy chain of Trastuzumab. Fluoroalkylation by Acetic Togni reagent (A) and by Acetic imidazole Togni reagent (B). Purple bars represent the extent of modification of Trastuzumab expired in August 2021, and green bars of Trastuzumab expired in January 2022. ***, P < 0.005; **, P < 0.01; *, P < 0.05

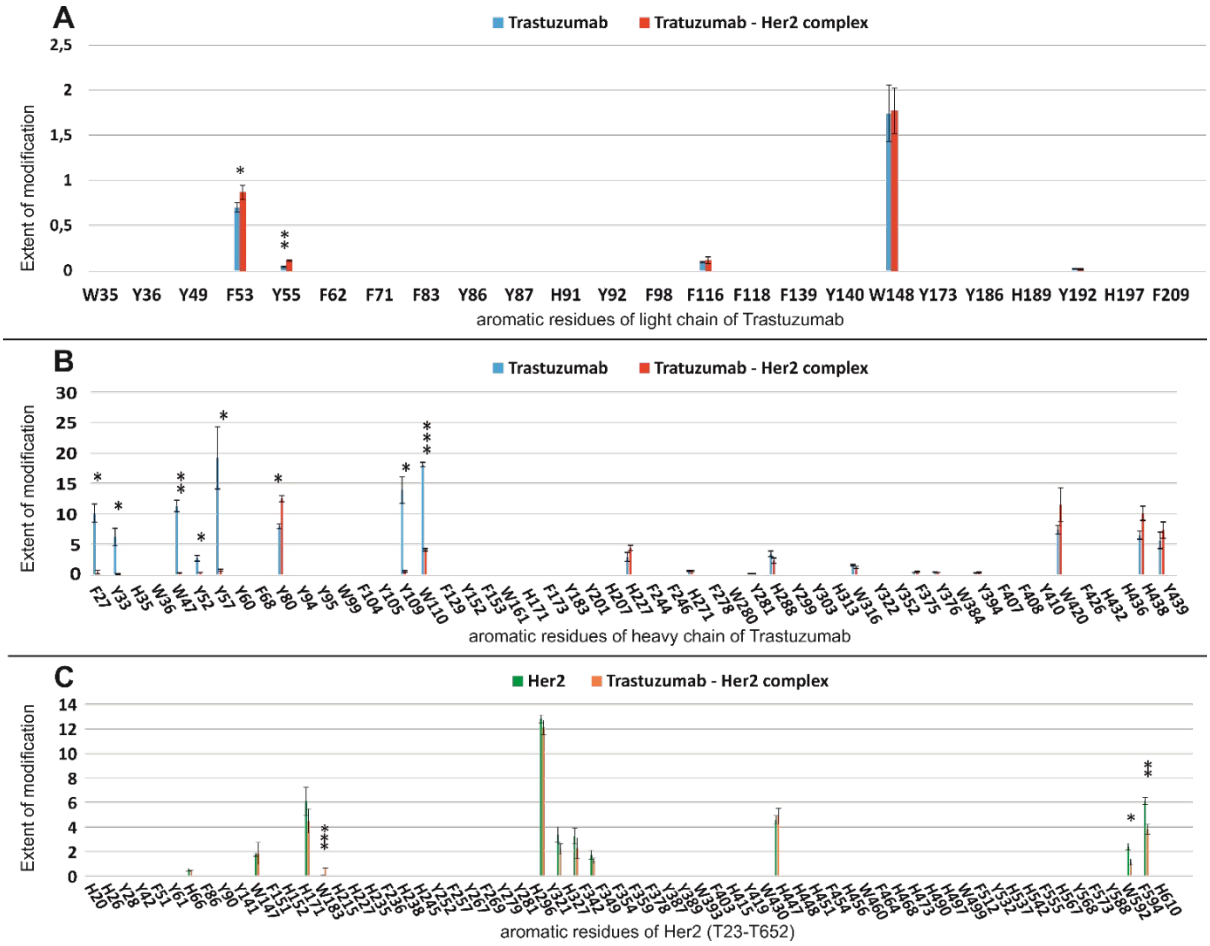

Figure S5 Quantification of the modification of the aromatic residues of Trastuzumab and complex of Trastuzumab with HER2. Aromatic residues of (A) light chain, (B) heavy chain of Trastuzumab and (C) HER2 modified by Acetic Imidazole Togni reagent for 3s labelling pulse. The extent of modification of Trastuzumab alone (blue bars), Trastuzumab in complex (red bars), HER2 alone (green bars) and HER2 in complex (orange bars). ***, P < 0.005; **, P < 0.01; *, P < 0.05.

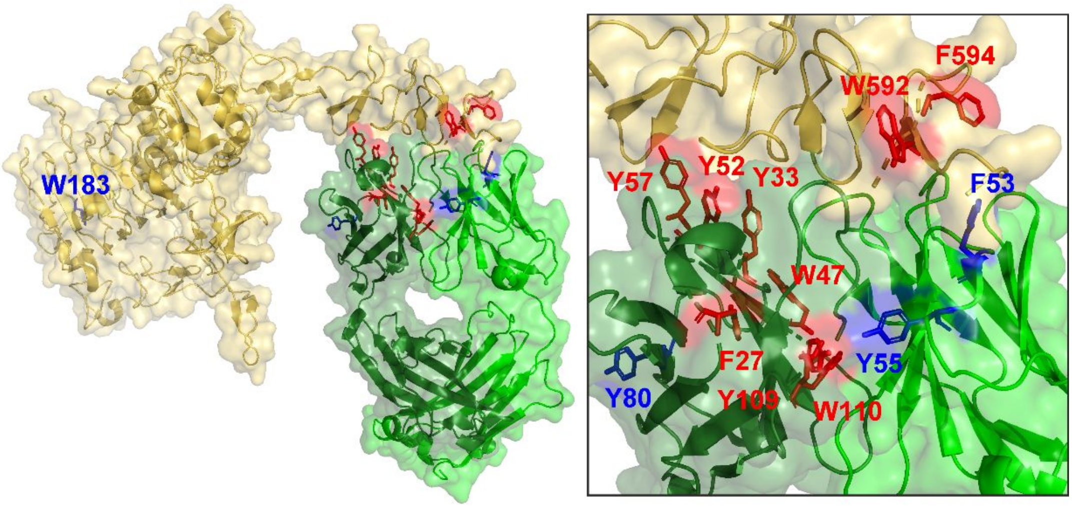

Figure S6 Structure of human complex HER2-Trastuzumab. Crystal structure of extracellular domain of human HER2 with Trastuzumab Fab with highlighted modified residues after reaction with Acetic imidazole Togni reagent Trastuzumab ligh chain (light green), Trustuzumab heavy chain (dark green) and HER2 (yellow) visualized on the crystal structure of complex (1N8Z). Red residues were less modified in complex whereas blue residues were more modified in the complex.

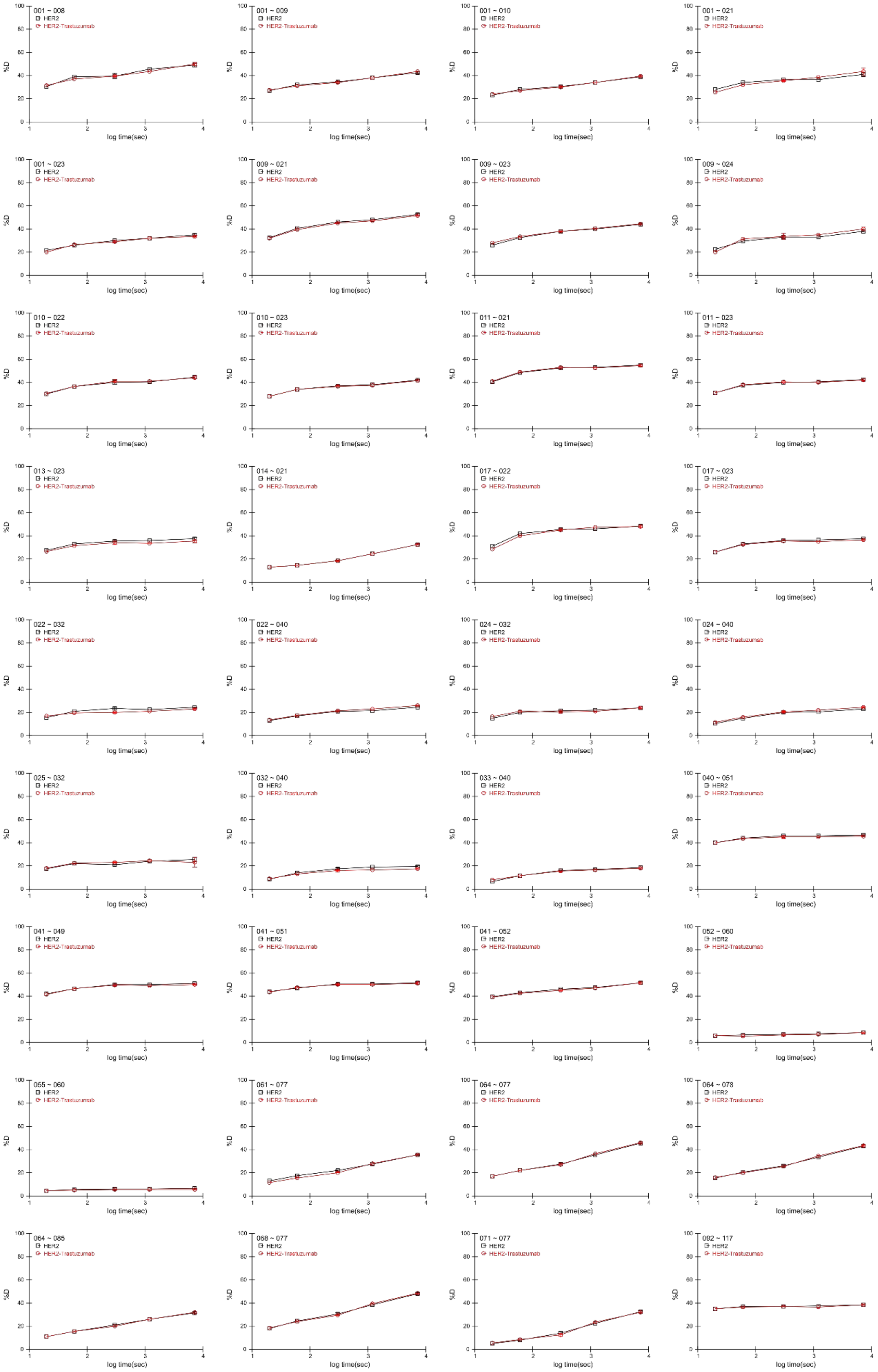
001 ~ 008
001 ~ 009
001 ~ 010
001 ~ 021
001 ~ 023
009 ~ 021
009 ~ 023
009 ~ 024
010 ~ 022
010 ~ 023
011 ~ 021
011 ~ 023
013 ~ 023
014 ~ 021
017 ~ 022
017 ~ 023
022 ~ 032
022 ~ 040
024 ~ 032
024 ~ 040
025 ~ 032
032 ~ 040
033 ~ 040
040 ~ 051
041 ~ 049
041 ~ 051
041 ~ 052
052 ~ 060
055 ~ 060
061 ~ 077
064 ~ 077
064 ~ 078
064 ~ 085
068 ~ 077
071 ~ 077
082 ~ 117
HER2
HER2-Trastuzumab
%D
log time(sec)

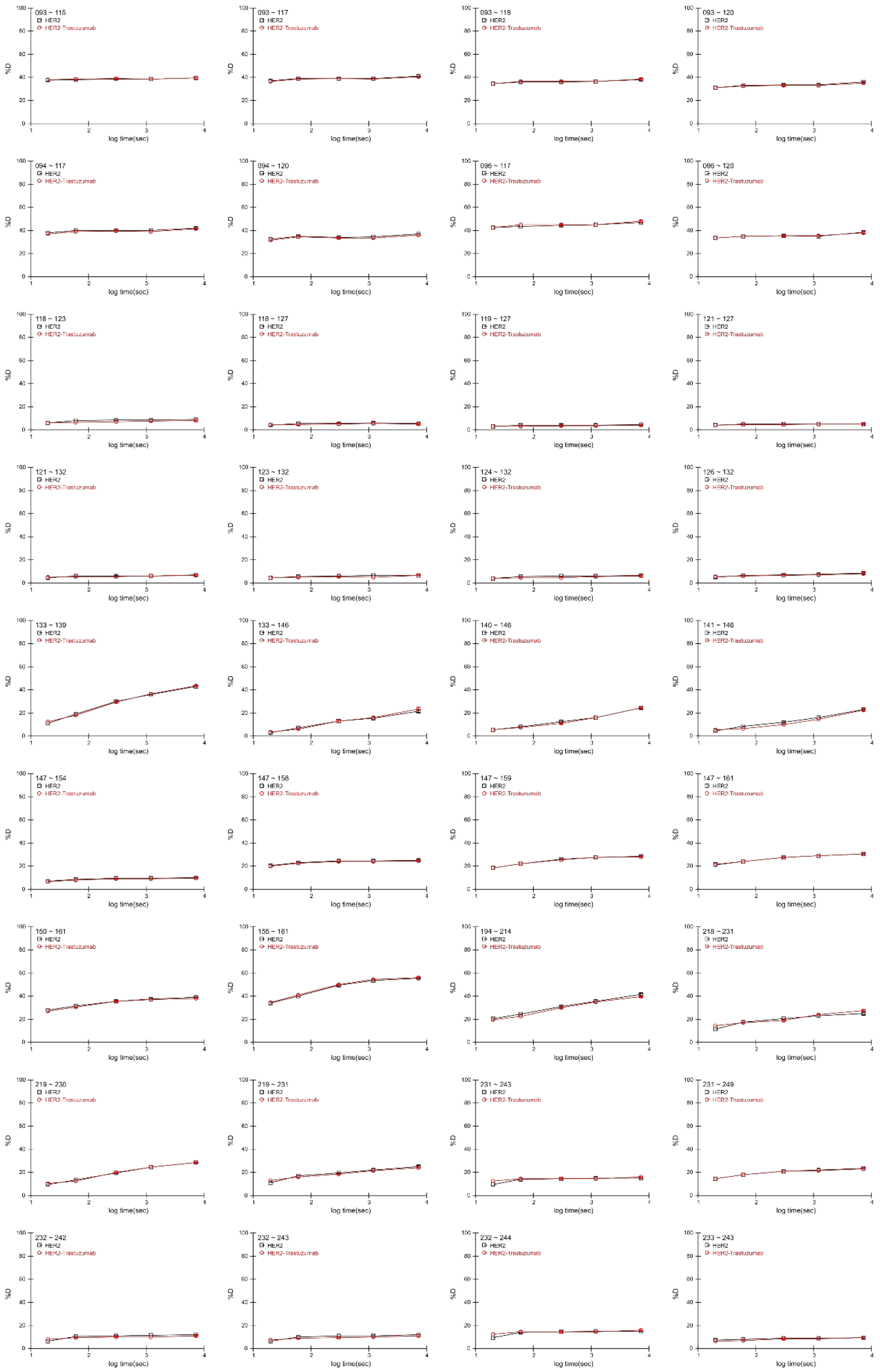
093 ~ 115
093 ~ 117
093 ~ 118
093 ~ 120
094 ~ 117
094 ~ 120
096 ~ 117
096 ~ 120
118 ~ 123
118 ~ 127
119 ~ 127
121 ~ 127
121 ~ 132
123 ~ 132
124 ~ 132
126 ~ 132
133 ~ 139
133 ~ 146
140 ~ 146
141 ~ 146
147 ~ 154
147 ~ 158
147 ~ 159
147 ~ 161
150 ~ 161
155 ~ 161
194 ~ 214
218 ~ 231
219 ~ 230
219 ~ 231
231 ~ 243
231 ~ 249
232 ~ 242
232 ~ 243
232 ~ 244
233 ~ 243
HER2
HER2-Trastuzumab
%D
log time(sec)

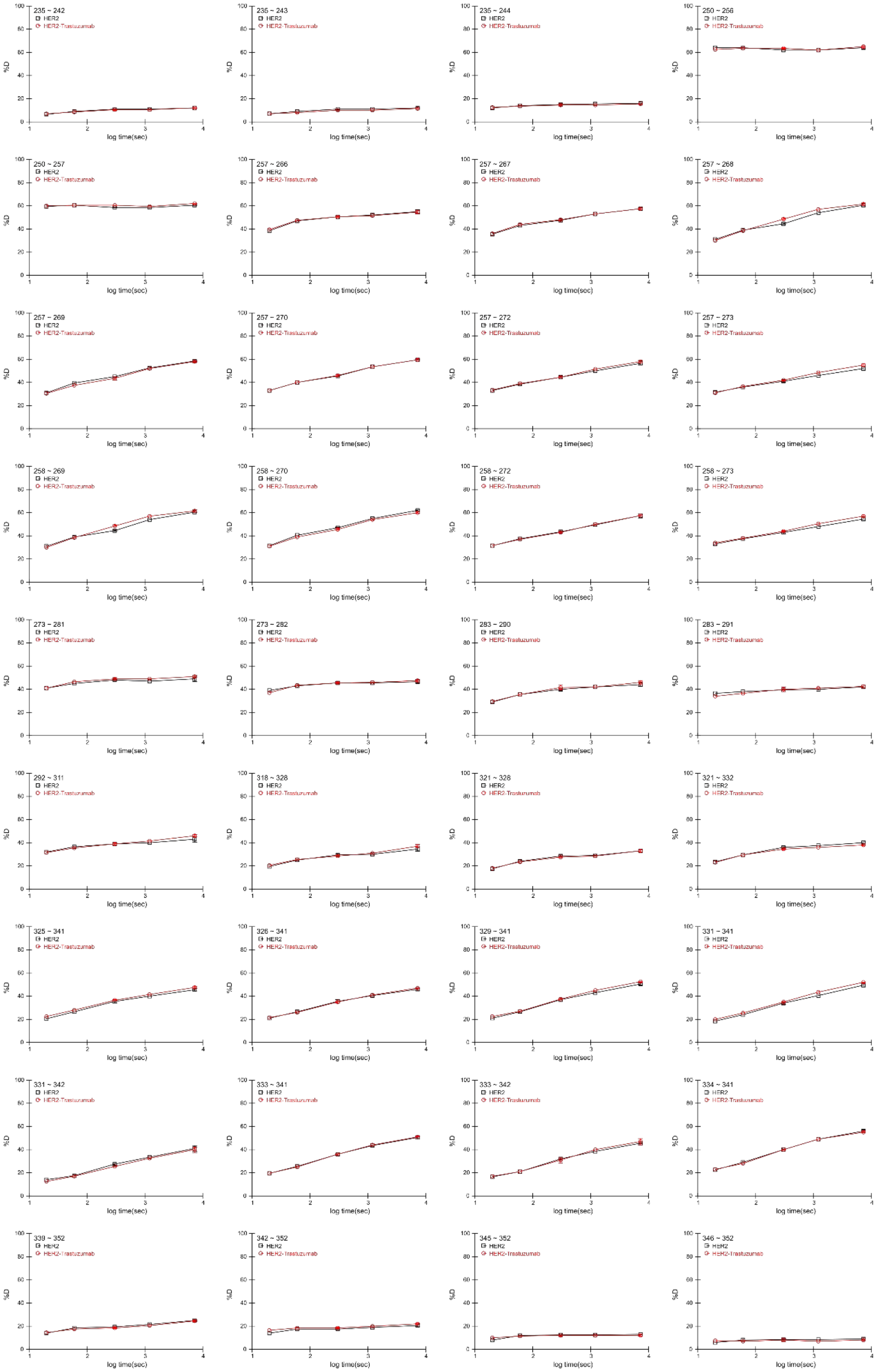
235 ~ 242
235 ~ 243
235 ~ 244
250 ~ 256
250 ~ 257
257 ~ 266
257 ~ 267
257 ~ 268
257 ~ 269
257 ~ 270
257 ~ 272
257 ~ 273
258 ~ 269
258 ~ 270
258 ~ 272
258 ~ 273
273 ~ 281
273 ~ 282
283 ~ 290
283 ~ 291
292 ~ 311
318 ~ 328
321 ~ 328
321 ~ 332
325 ~ 341
326 ~ 341
329 ~ 341
331 ~ 341
331 ~ 342
333 ~ 341
333 ~ 342
334 ~ 341
339 ~ 352
342 ~ 352
345 ~ 352
346 ~ 352
HER2
HER2-Trastuzumab
%D
log time(sec)

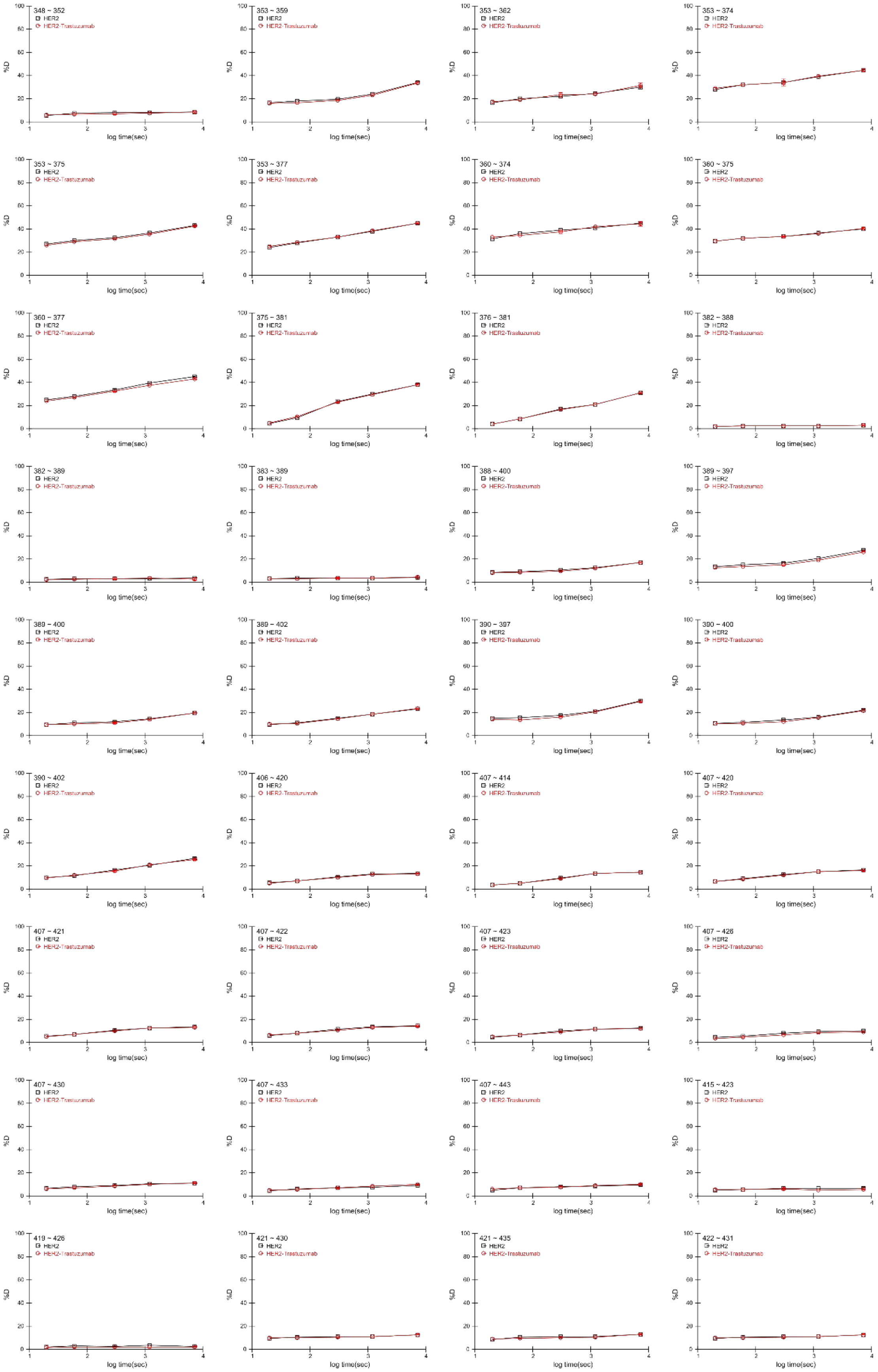
348 ~ 352
353 ~ 359
353 ~ 362
353 ~ 374
353 ~ 375
353 ~ 377
360 ~ 374
360 ~ 375
360 ~ 377
375 ~ 381
376 ~ 381
382 ~ 388
382 ~ 389
383 ~ 389
388 ~ 400
389 ~ 397
389 ~ 400
389 ~ 402
390 ~ 397
390 ~ 400
390 ~ 402
406 ~ 420
407 ~ 414
407 ~ 420
407 ~ 421
407 ~ 422
407 ~ 423
407 ~ 426
407 ~ 430
407 ~ 433
407 ~ 443
415 ~ 423
419 ~ 426
421 ~ 430
421 ~ 435
422 ~ 431
HER2
HER2-Trastuzumab
%D
log time(sec)

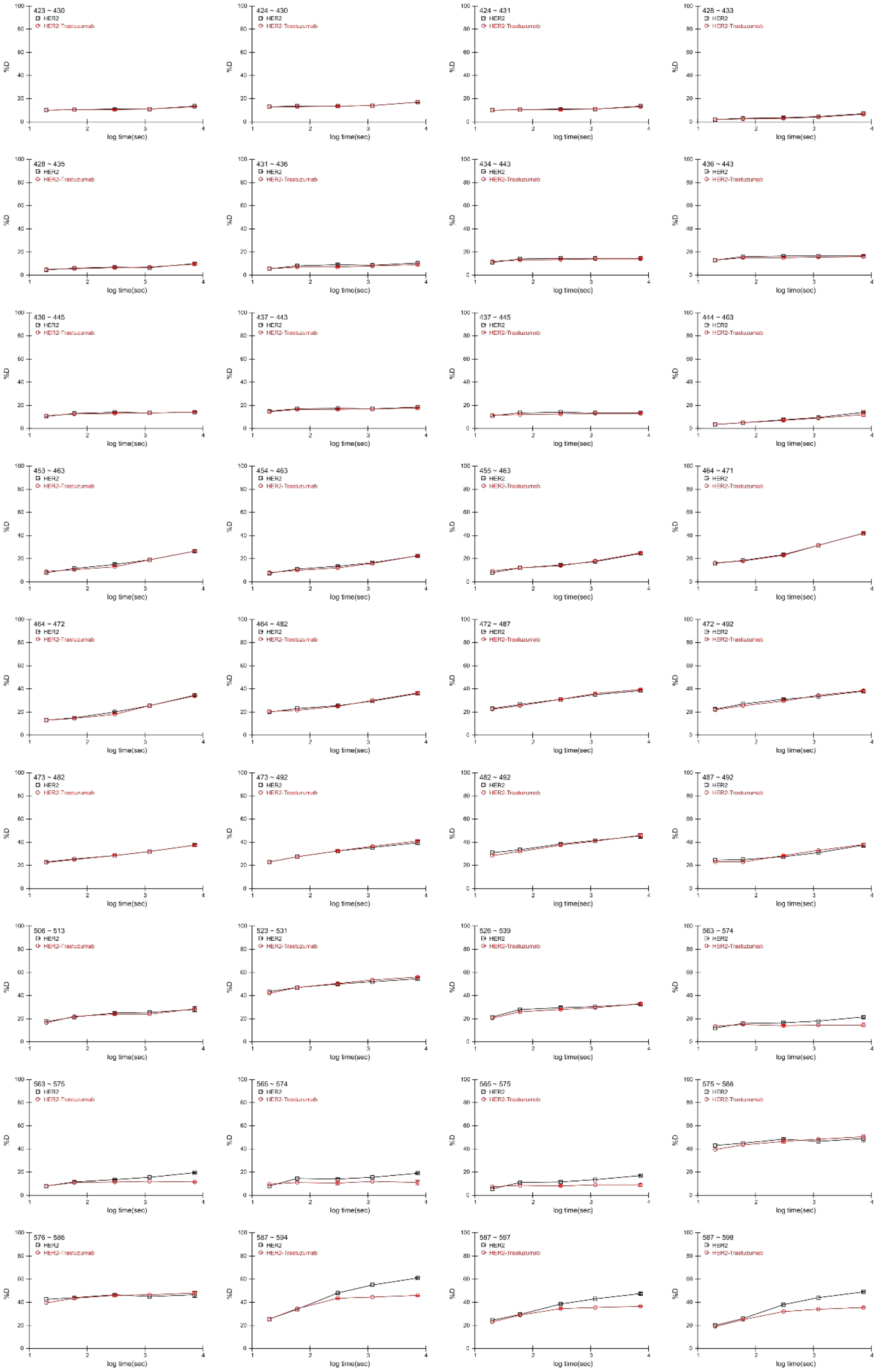
423 ~ 430
424 ~ 430
424 ~ 431
428 ~ 433
428 ~ 435
431 ~ 436
434 ~ 443
436 ~ 443
436 ~ 445
437 ~ 443
437 ~ 445
444 ~ 463
453 ~ 463
454 ~ 463
455 ~ 463
464 ~ 471
464 ~ 472
464 ~ 482
472 ~ 487
472 ~ 492
473 ~ 482
473 ~ 492
482 ~ 492
487 ~ 492
506 ~ 513
523 ~ 531
526 ~ 539
563 ~ 574
563 ~ 575
565 ~ 574
565 ~ 575
575 ~ 586
576 ~ 586
587 ~ 594
587 ~ 597
587 ~ 598
HER2
HER2-Trastuzumab
%D
log time(sec)

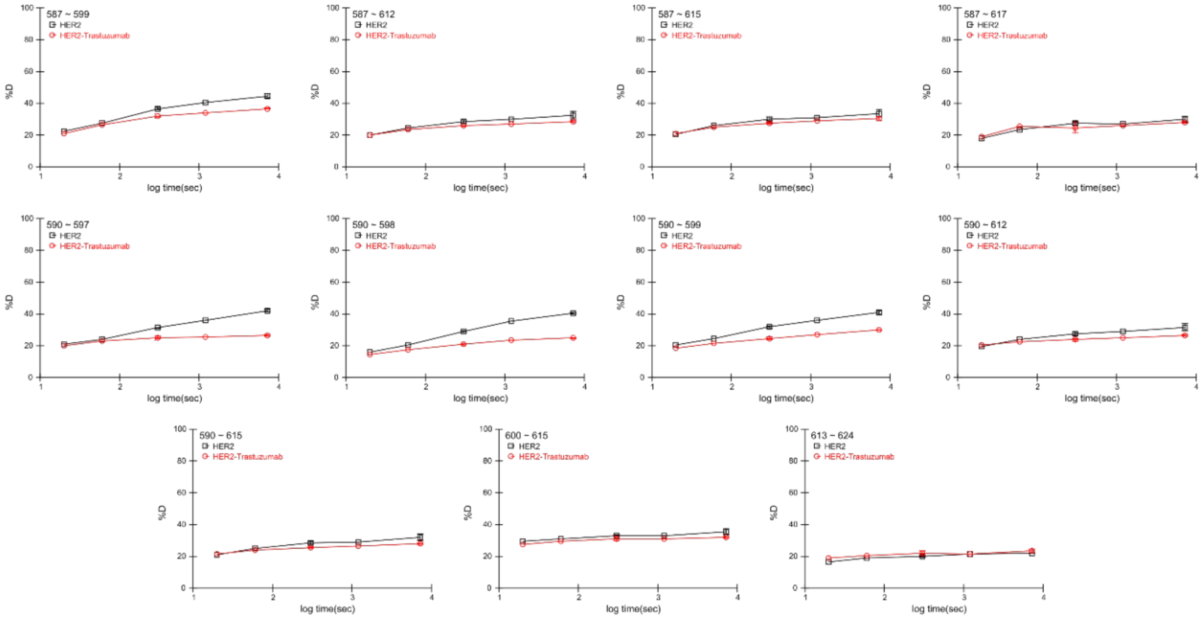

Figure S7. HER2-Trastuzumab interaction probed by HDX-MS - Deuterium uptake plots of human HER2 peptides at two different states: alone (black) or in complex with Trastuzumab (red). Percentage of deuteration as a function of time.

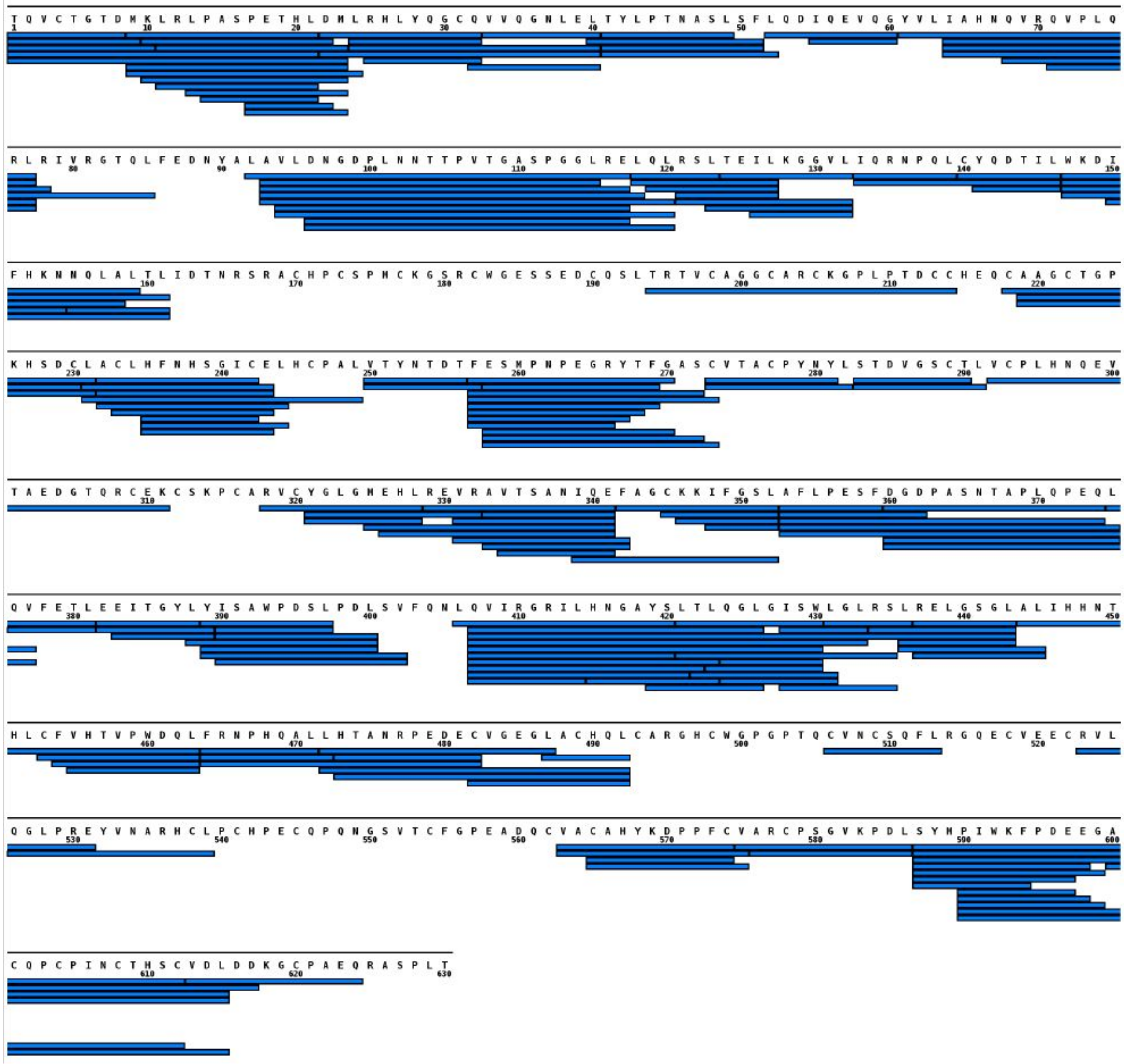

Figure S8. Sequence coverage of HER2 in HDX-MS. Overview of the sequence coverage by the peptides providing HDX readout. Digestion was done on a co-immobilized pepsin/nepenthesin-2 column coupled with online deglycosylation by PNGaseRc column. Final sequence coverage of 84 % was achieved by 191 peptides with an average length of 12.9 amino acid residues and a redundancy score of 3.9. peptides are represented by the blue bars. The map was created using the DrawMap tool, part of MSTools - http://peterslab.org/MSTools/DrawMap/DrawMap.php.

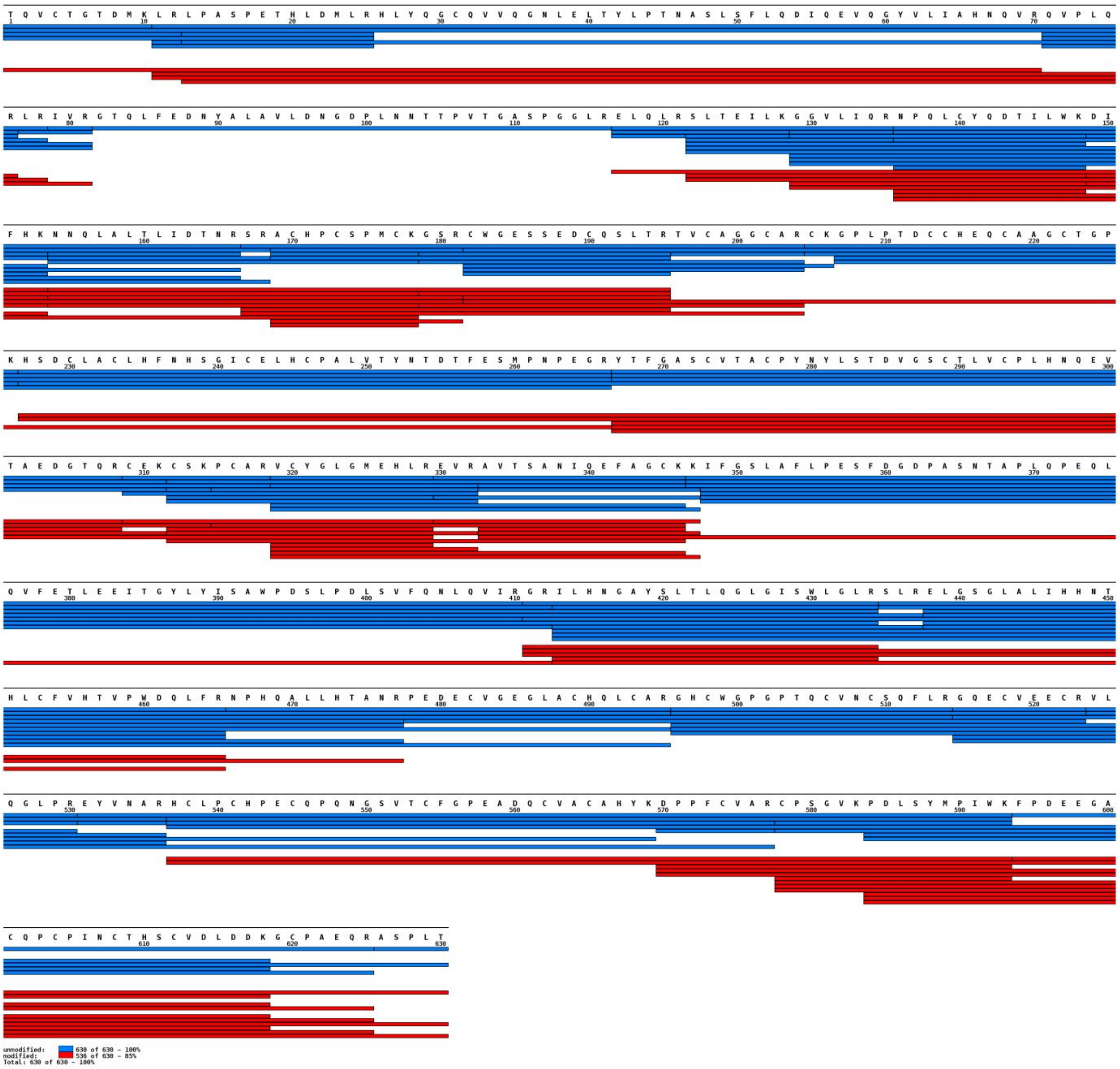

Figure S9. Sequence coverage of HER2 probed by FFAP using the Acetic Togni reagent. Overview of the sequence coverage by unmodified peptides represented by the blue bars and peptides modified by Acetic Togni reagents represented by the red bars. Final sequence coverage of 85 % was achieved by 61 modified peptides with an average length of 38.4 amino acid residues and a redundancy score of 3.7. The map was created using the DrawMap tool, part of MSTools - http://peterslab.org/MSTools/DrawMap/DrawMap.php.

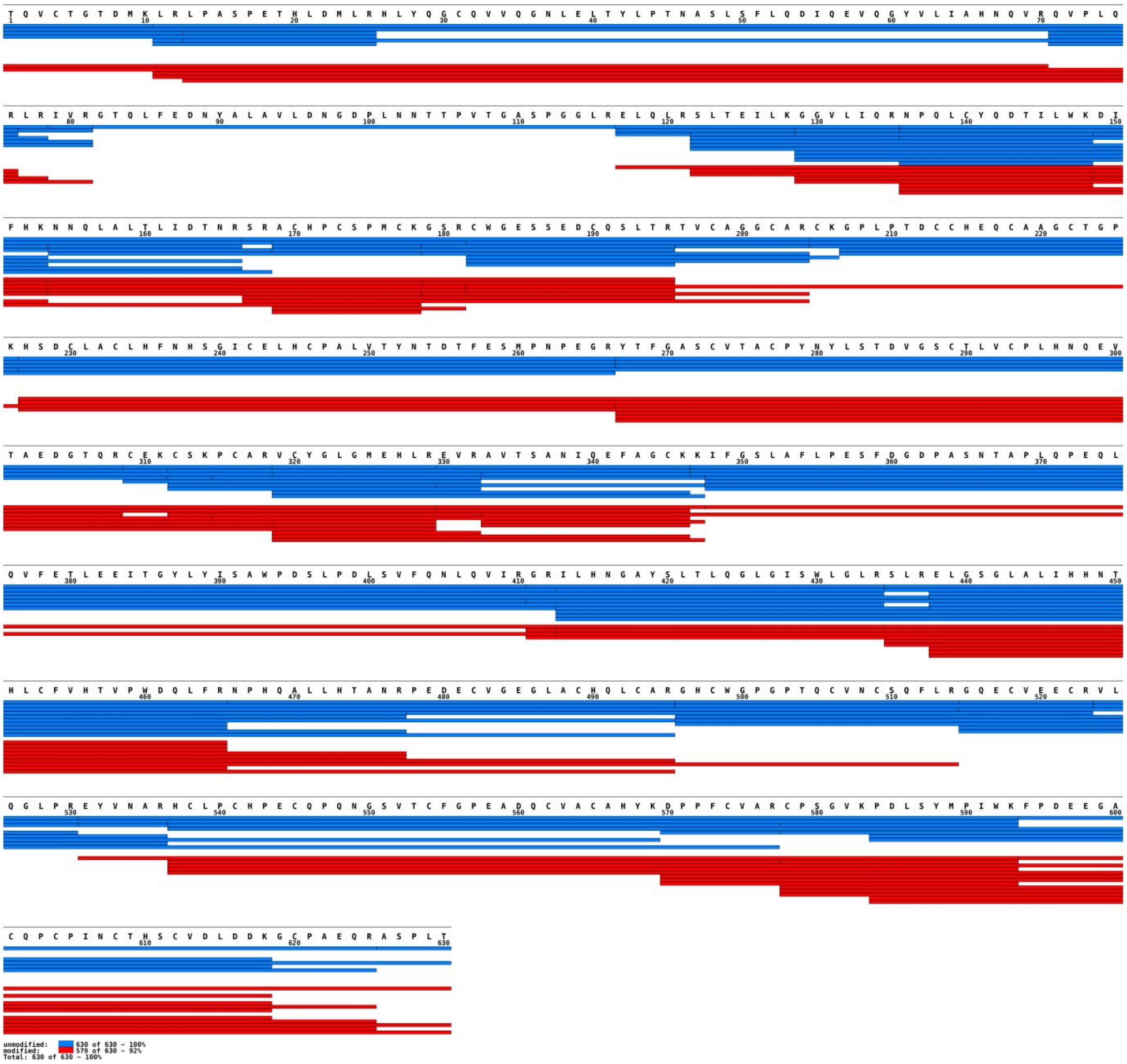

Figure S10. Sequence coverage of HER2 probed by FFAP using the Acetic Imidazole Togni reagent. Overview of the sequence coverage by unmodified peptides represented by the blue bars and peptides modified by Acetic Imidazole Togni reagents represented by the red bars. Final sequence coverage of 92 % was achieved by 73 modified peptides with an average length of 41.7 amino acid residues and a redundancy score of 4.8. The map was created using the DrawMap tool, part of MSTools - http://peterslab.org/MSTools/DrawMap/DrawMap.php.

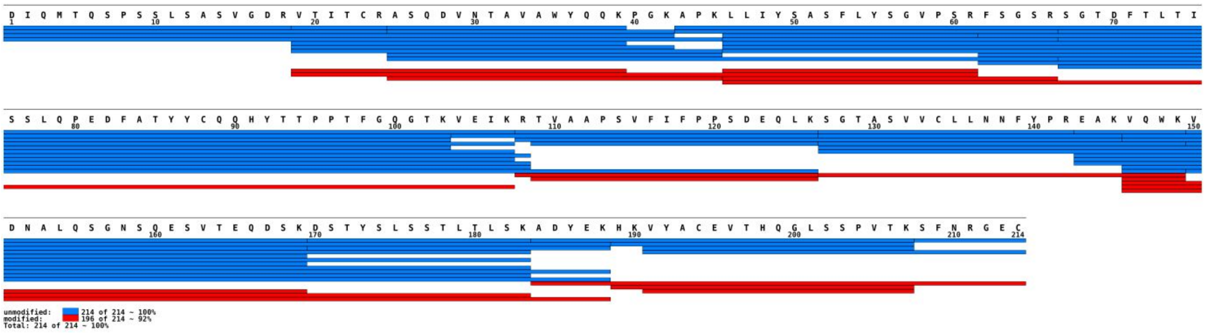

Figure S11. Sequence coverage of Trastuzumab light chain probed by FFAP using the Acetic Togni reagent. Overview of the sequence coverage by unmodified peptides represented by the blue bars and peptides modified by Acetic Togni reagents represented by the red bars. Final sequence coverage of 92 % was achieved by 16 modified peptides with an average length of 26.2 amino acid residues and a redundancy score of 2.0. The map was created using the DrawMap tool, part of MSTools - http://peterslab.org/MSTools/DrawMap/DrawMap.php.

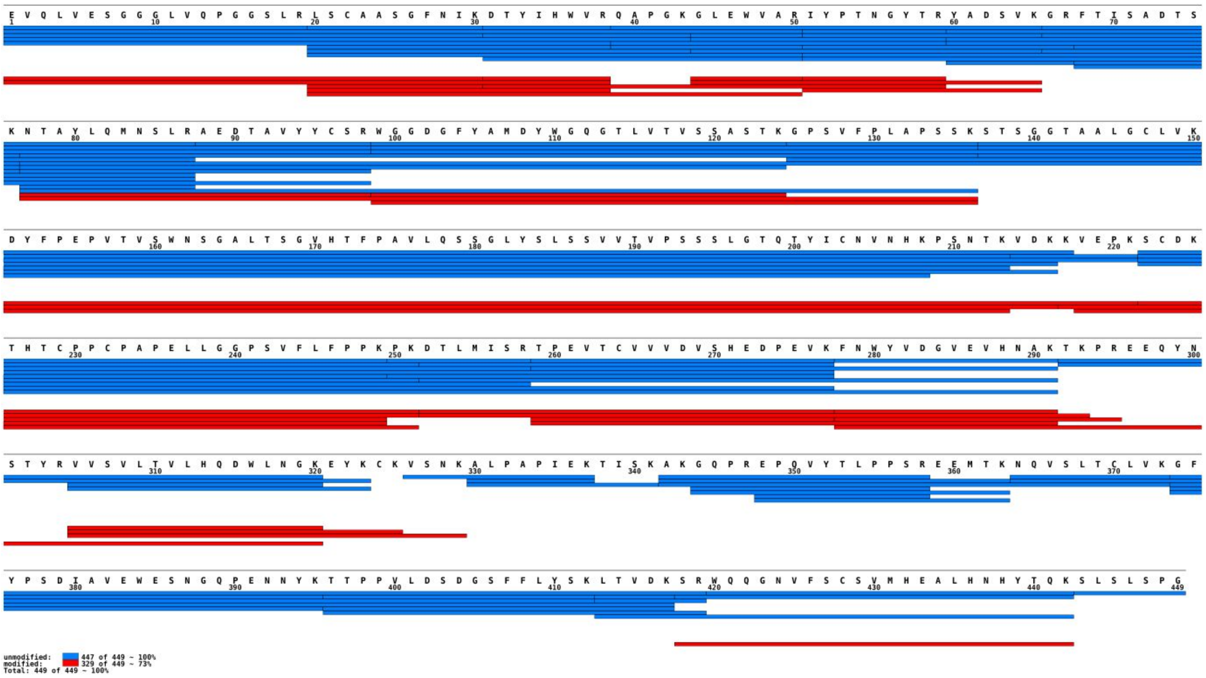

Figure S12. Sequence coverage of Trastuzumab heavy chain probed by FFAP using the Acetic Togni reagent. Overview of the sequence coverage by unmodified peptides represented by the blue bars and peptides modified by Acetic Togni reagents represented by the red bars. Final sequence coverage of 73 % was achieved by 34 modified peptides with an average length of 29.2 amino acid residues and a redundancy score of 2.2. The map was created using the DrawMap tool, part of MSTools - http://peterslab.org/MSTools/DrawMap/DrawMap.php.

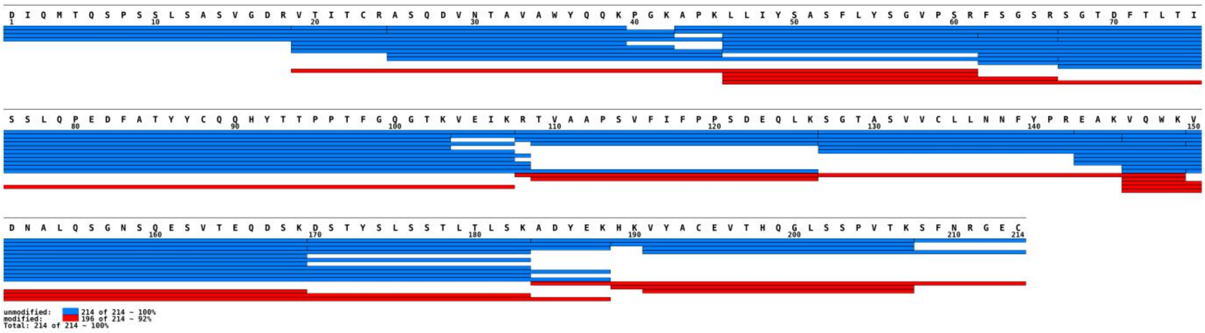

Figure S13. Sequence coverage of Trastuzumab light chain probed by FFAP using Acetic Imidazole Togni reagent. Overview of the sequence coverage by unmodified peptides represented by the blue bars and peptides modified by Acetic Imidazole Togni reagents represented by the red bars. Final sequence coverage of 92 % was achieved by 14 modified peptides with an average length of 27.0 amino acid residues and a redundancy score of 1.8. The map was created using the DrawMap tool, part of MSTools - http://peterslab.org/MSTools/DrawMap/DrawMap.php.

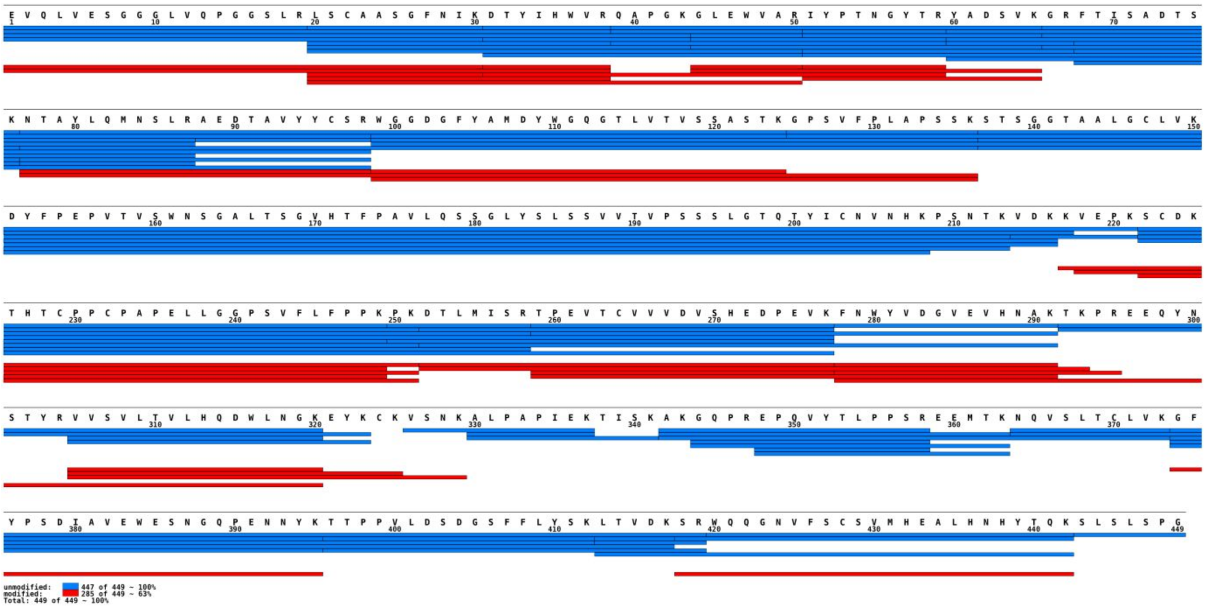

Figure S14. Sequence coverage of Trastuzumab heavy chain probed by FFAP using Acetic Imidazole Togni reagent. Overview of the sequence coverage by unmodified peptides represented by the blue bars and peptides modified by Acetic Imidazole Togni reagents represented by the red bars. Final sequence coverage of 63 % was achieved by 32 modified peptides with an average length of 25.5 amino acid residues and a redundancy score of 1.8. The map was created using the DrawMap tool, part of MSTools - http://peterslab.org/MSTools/DrawMap/DrawMap.php.

Table S1 Table of surface-accessible solvent area for CF_3 radical for crystal structure of extracellular domain of human HER2 with Trastuzumab Fab (1N8Z). Absolute values represent accessible surface area in square angstroms for all atoms of each residue (columns on the left) or only for side chain atoms of residue (columns on the right). Relative values are related to accessibility of theoretic residue in tripeptide Ala-x-ALA. Not applicable (N/A) values belongs to residues that are missing in the crystal structure.

Molecule	Residue	Number	Area of all atoms		Area of side chain residues	
			Absolute Å²	Relative %	Absolute Å²	Relative %
Ab-Light Chain	ASP	1	102.67	73.1	60.99	59.4
Ab-Light Chain	ILE	2	0	0	0	0
Ab-Light Chain	GLN	3	115.2	64.5	115.2	81.7
Ab-Light Chain	MET	4	3.73	1.9	0	0
Ab-Light Chain	THR	5	69.88	50.2	69.88	68.7
Ab-Light Chain	GLN	6	0.91	0.5	0	0
Ab-Light Chain	SER	7	51.35	44.1	51.35	65.7
Ab-Light Chain	PRO	8	57.32	42.1	57.32	47.8
Ab-Light Chain	SER	9	82.02	70.4	74.59	95.5
Ab-Light Chain	SER	10	60.76	52.2	44.98	57.6
Ab-Light Chain	LEU	11	22.36	12.5	22.36	15.8
Ab-Light Chain	SER	12	50.43	43.3	26.36	33.8
Ab-Light Chain	ALA	13	3.35	3.1	3.35	4.8
Ab-Light Chain	SER	14	41.82	35.9	41.82	53.5
Ab-Light Chain	VAL	15	84.44	55.8	61.75	54
Ab-Light Chain	GLY	16	54.61	68.2	27.2	84.1
Ab-Light Chain	ASP	17	69.5	49.5	69.5	67.7
Ab-Light Chain	ARG	18	195.32	81.8	176.74	87.8
Ab-Light Chain	VAL	19	3.73	2.5	3.73	3.3
Ab-Light Chain	THR	20	61.66	44.3	57.34	56.4
Ab-Light Chain	ILE	21	0	0	0	0
Ab-Light Chain	THR	22	22.84	16.4	22.84	22.5
Ab-Light Chain	CYS	23	0	0	0	0
Ab-Light Chain	ARG	24	150.94	63.2	150.94	75
Ab-Light Chain	ALA	25	0.45	0.4	0	0
Ab-Light Chain	SER	26	59.18	50.8	23.42	30
Ab-Light Chain	GLN	27	98.63	55.3	98.63	70
Ab-Light Chain	ASP	28	58.88	41.9	54.44	53
Ab-Light Chain	VAL	29	0	0	0	0
Ab-Light Chain	ASN	30	0	0	0	0
Ab-Light Chain	THR	31	11.92	8.6	11.92	11.7
Ab-Light Chain	ALA	32	0	0	0	0
Ab-Light Chain	VAL	33	0	0	0	0

Ab-Light Chain	ALA	34	0	0	0	0
Ab-Light Chain	TRP	35	0	0	0	0
Ab-Light Chain	TYR	36	0	0	0	0
Ab-Light Chain	GLN	37	6.46	3.6	6.46	4.6
Ab-Light Chain	GLN	38	4.35	2.4	4.35	3.1
Ab-Light Chain	LYS	39	36.01	17.9	35.88	22
Ab-Light Chain	PRO	40	41.31	30.3	35.3	29.4
Ab-Light Chain	GLY	41	77.58	96.9	47.99	148.4
Ab-Light Chain	LYS	42	123.91	61.7	123.91	75.9
Ab-Light Chain	ALA	43	4.44	4.1	4.15	6
Ab-Light Chain	PRO	44	0	0	0	0
Ab-Light Chain	LYS	45	110.67	55.1	110.67	67.8
Ab-Light Chain	LEU	46	0	0	0	0
Ab-Light Chain	LEU	47	0.95	0.5	0.95	0.7
Ab-Light Chain	ILE	48	0	0	0	0
Ab-Light Chain	TYR	49	30.65	14.4	30.65	17.3
Ab-Light Chain	SER	50	0	0	0	0
Ab-Light Chain	ALA	51	0.11	0.1	0.11	0.2
Ab-Light Chain	SER	52	57.36	49.2	38.74	49.6
Ab-Light Chain	PHE	53	32.32	16.2	32.32	19.7
Ab-Light Chain	LEU	54	61.24	34.3	46.53	33
Ab-Light Chain	TYR	55	26.82	12.6	26.6	15
Ab-Light Chain	SER	56	113.93	97.8	101.76	130.3
Ab-Light Chain	GLY	57	99.85	124.7	53.77	166.3
Ab-Light Chain	VAL	58	12.08	8	5.86	5.1
Ab-Light Chain	PRO	59	56.04	41.2	56.04	46.7
Ab-Light Chain	SER	60	118.35	101.6	100.59	128.8
Ab-Light Chain	ARG	61	33.69	14.1	33.69	16.7
Ab-Light Chain	PHE	62	0.58	0.3	0.27	0.2
Ab-Light Chain	SER	63	47.64	40.9	47.64	61
Ab-Light Chain	GLY	64	3.8	4.7	0	0
Ab-Light Chain	SER	65	55.01	47.2	55.01	70.4
Ab-Light Chain	ARG	66	75.86	31.8	51.2	25.4
Ab-Light Chain	SER	67	96.74	83	88.58	113.4
Ab-Light Chain	GLY	68	55.87	69.7	44.15	136.6
Ab-Light Chain	THR	69	43.07	30.9	42.18	41.5
Ab-Light Chain	ASP	70	29.43	21	29.43	28.7
Ab-Light Chain	PHE	71	0	0	0	0
Ab-Light Chain	THR	72	18.91	13.6	18.91	18.6
Ab-Light Chain	LEU	73	0	0	0	0
Ab-Light Chain	THR	74	1.22	0.9	1.22	1.2

Ab-Light Chain	ILE	75	0	0	0	0
Ab-Light Chain	SER	76	51.65	44.3	43.06	55.1
Ab-Light Chain	SER	77	47.5	40.8	47.5	60.8
Ab-Light Chain	LEU	78	0	0	0	0
Ab-Light Chain	GLN	79	50.23	28.1	50.23	35.6
Ab-Light Chain	PRO	80	21.89	16.1	21.89	18.3
Ab-Light Chain	GLU	81	68.25	39.6	68.25	50.7
Ab-Light Chain	ASP	82	0	0	0	0
Ab-Light Chain	PHE	83	0	0	0	0
Ab-Light Chain	ALA	84	0	0	0	0
Ab-Light Chain	THR	85	21.36	15.3	21.36	21
Ab-Light Chain	TYR	86	0	0	0	0
Ab-Light Chain	TYR	87	2.14	1	2.14	1.2
Ab-Light Chain	CYS	88	0	0	0	0
Ab-Light Chain	GLN	89	0	0	0	0
Ab-Light Chain	GLN	90	0	0	0	0
Ab-Light Chain	HIS	91	0	0	0	0
Ab-Light Chain	TYR	92	28.62	13.5	28.62	16.1
Ab-Light Chain	THR	93	37.07	26.6	37.07	36.5
Ab-Light Chain	THR	94	12.46	8.9	0	0
Ab-Light Chain	PRO	95	8.83	6.5	8.83	7.4
Ab-Light Chain	PRO	96	0	0	0	0
Ab-Light Chain	THR	97	22.79	16.4	22.79	22.4
Ab-Light Chain	PHE	98	16.53	8.3	0.1	0.1
Ab-Light Chain	GLY	99	0	0	0	0
Ab-Light Chain	GLN	100	135.24	75.8	124.95	88.6
Ab-Light Chain	GLY	101	0.97	1.2	0	0
Ab-Light Chain	THR	102	0	0	0	0
Ab-Light Chain	LYS	103	41.86	20.8	41.86	25.6
Ab-Light Chain	VAL	104	0	0	0	0
Ab-Light Chain	GLU	105	45.62	26.5	45.62	33.9
Ab-Light Chain	ILE	106	0	0	0	0
Ab-Light Chain	LYS	107	110.38	55	110.36	67.6
Ab-Light Chain	ARG	108	79.78	33.4	62.65	31.1
Ab-Light Chain	THR	109	119.84	86	119.6	117.6
Ab-Light Chain	VAL	110	82.86	54.7	70.58	61.8
Ab-Light Chain	ALA	111	13.57	12.6	13.57	19.6
Ab-Light Chain	ALA	112	55.52	51.4	41.21	59.4
Ab-Light Chain	PRO	113	4.48	3.3	0	0
Ab-Light Chain	SER	114	37.26	32	37.26	47.7
Ab-Light Chain	VAL	115	2.52	1.7	0.02	0

Ab-Light Chain	PHE	116	0	0	0	0
Ab-Light Chain	ILE	117	0	0	0	0
Ab-Light Chain	PHE	118	0	0	0	0
Ab-Light Chain	PRO	119	7.22	5.3	7.22	6
Ab-Light Chain	PRO	120	3.57	2.6	0	0
Ab-Light Chain	SER	121	3.32	2.9	3.32	4.3
Ab-Light Chain	ASP	122	136.27	97.1	134.29	130.8
Ab-Light Chain	GLU	123	77.9	45.2	77.6	57.6
Ab-Light Chain	GLN	124	0	0	0	0
Ab-Light Chain	LEU	125	12.85	7.2	12.85	9.1
Ab-Light Chain	LYS	126	163.36	81.4	128.7	78.8
Ab-Light Chain	SER	127	92.24	79.2	59.91	76.7
Ab-Light Chain	GLY	128	31.7	39.6	25.9	80.1
Ab-Light Chain	THR	129	29.27	21	29.27	28.8
Ab-Light Chain	ALA	130	0	0	0	0
Ab-Light Chain	SER	131	0	0	0	0
Ab-Light Chain	VAL	132	0	0	0	0
Ab-Light Chain	VAL	133	0	0	0	0
Ab-Light Chain	CYS	134	0	0	0	0
Ab-Light Chain	LEU	135	0	0	0	0
Ab-Light Chain	LEU	136	0	0	0	0
Ab-Light Chain	ASN	137	0	0	0	0
Ab-Light Chain	ASN	138	38.79	26.9	36.71	34.6
Ab-Light Chain	PHE	139	0	0	0	0
Ab-Light Chain	TYR	140	8.8	4.1	8.8	5
Ab-Light Chain	PRO	141	28.9	21.2	28.9	24.1
Ab-Light Chain	ARG	142	115.51	48.4	115.25	57.3
Ab-Light Chain	GLU	143	151.84	88.2	150.03	111.3
Ab-Light Chain	ALA	144	8.46	7.8	0.77	1.1
Ab-Light Chain	LYS	145	122.39	60.9	122.39	74.9
Ab-Light Chain	VAL	146	14.92	9.9	0	0
Ab-Light Chain	GLN	147	34.72	19.5	34.72	24.6
Ab-Light Chain	TRP	148	0	0	0	0
Ab-Light Chain	LYS	149	55.29	27.5	55.29	33.9
Ab-Light Chain	VAL	150	0	0	0	0
Ab-Light Chain	ASP	151	58.88	41.9	40.98	39.9
Ab-Light Chain	ASN	152	134.01	93.1	108.27	101.9
Ab-Light Chain	ALA	153	60	55.6	60	86.4
Ab-Light Chain	LEU	154	123.39	69.1	104.09	73.8
Ab-Light Chain	GLN	155	31.09	17.4	21.81	15.5
Ab-Light Chain	SER	156	111.29	95.5	84.24	107.8

Ab-Light Chain	GLY	157	95.35	119	51.95	160.7
Ab-Light Chain	ASN	158	25.75	17.9	25.25	23.8
Ab-Light Chain	SER	159	26.67	22.9	9.11	11.7
Ab-Light Chain	GLN	160	49.01	27.5	49.01	34.8
Ab-Light Chain	GLU	161	70.33	40.8	52.32	38.8
Ab-Light Chain	SER	162	1.53	1.3	1.53	2
Ab-Light Chain	VAL	163	9.47	6.3	9.21	8.1
Ab-Light Chain	THR	164	4.65	3.3	4.65	4.6
Ab-Light Chain	GLU	165	58.89	34.2	55.64	41.3
Ab-Light Chain	GLN	166	1.78	1	0	0
Ab-Light Chain	ASP	167	26.51	18.9	26.51	25.8
Ab-Light Chain	SER	168	38.02	32.6	36.65	46.9
Ab-Light Chain	LYS	169	176.9	88.1	162.24	99.4
Ab-Light Chain	ASP	170	59.44	42.3	58.84	57.3
Ab-Light Chain	SER	171	0.24	0.2	0.24	0.3
Ab-Light Chain	THR	172	0.92	0.7	0.92	0.9
Ab-Light Chain	TYR	173	2.31	1.1	2.31	1.3
Ab-Light Chain	SER	174	0	0	0	0
Ab-Light Chain	LEU	175	0.12	0.1	0.12	0.1
Ab-Light Chain	SER	176	0.02	0	0.02	0
Ab-Light Chain	SER	177	0	0	0	0
Ab-Light Chain	THR	178	0	0	0	0
Ab-Light Chain	LEU	179	0	0	0	0
Ab-Light Chain	THR	180	33.37	24	26.48	26
Ab-Light Chain	LEU	181	5.66	3.2	5.66	4
Ab-Light Chain	SER	182	55.61	47.7	50.24	64.3
Ab-Light Chain	LYS	183	77.79	38.7	77.79	47.6
Ab-Light Chain	ALA	184	78.45	72.7	77.46	111.6
Ab-Light Chain	ASP	185	68.64	48.9	68.64	66.8
Ab-Light Chain	TYR	186	0.11	0.1	0.11	0.1
Ab-Light Chain	GLU	187	78.03	45.3	66.57	49.4
Ab-Light Chain	LYS	188	166.1	82.7	131.11	80.3
Ab-Light Chain	HIS	189	46.94	25.7	46.93	31.9
Ab-Light Chain	LYS	190	50.37	25.1	49.54	30.3
Ab-Light Chain	VAL	191	41.58	27.5	41.58	36.4
Ab-Light Chain	TYR	192	0	0	0	0
Ab-Light Chain	ALA	193	0.66	0.6	0.66	1
Ab-Light Chain	CYS	194	0	0	0	0
Ab-Light Chain	GLU	195	31.39	18.2	31.39	23.3
Ab-Light Chain	VAL	196	0	0	0	0
Ab-Light Chain	THR	197	32.03	23	30.77	30.3

Ab-Light Chain	HIS	198	1.42	0.8	1.42	1
Ab-Light Chain	GLN	199	119.5	66.9	91.04	64.6
Ab-Light Chain	GLY	200	19.09	23.8	0.56	1.7
Ab-Light Chain	LEU	201	25.98	14.5	17.67	12.5
Ab-Light Chain	SER	202	154.18	132.3	119.66	153.2
Ab-Light Chain	SER	203	96.69	83	94.49	121
Ab-Light Chain	PRO	204	54.98	40.4	43.26	36.1
Ab-Light Chain	VAL	205	44.67	29.5	44.67	39.1
Ab-Light Chain	THR	206	84.68	60.8	74.28	73
Ab-Light Chain	LYS	207	32.23	16	32.23	19.7
Ab-Light Chain	SER	208	26.33	22.6	23.43	30
Ab-Light Chain	PHE	209	1.27	0.6	1.27	0.8
Ab-Light Chain	ASN	210	61.53	42.7	58.32	54.9
Ab-Light Chain	ARG	211	80.18	33.6	70.6	35.1
Ab-Light Chain	GLY	212	70.78	88.4	34.68	107.3
Ab-Light Chain	GLU	213	155.33	90.2	130.8	97.1
Ab-Light Chain	CYS	214	169.54	126.3	140.18	144.9
Ab-Heavy Chain	GLU	1	207.86	120.7	130.21	96.6
Ab-Heavy Chain	VAL	2	17.97	11.9	16.08	14.1
Ab-Heavy Chain	GLN	3	89.45	50.1	89.45	63.4
Ab-Heavy Chain	LEU	4	0	0	0	0
Ab-Heavy Chain	VAL	5	64.69	42.7	64.69	56.6
Ab-Heavy Chain	GLU	6	3.2	1.9	0	0
Ab-Heavy Chain	SER	7	58.26	50	58.26	74.6
Ab-Heavy Chain	GLY	8	22.31	27.8	21.19	65.5
Ab-Heavy Chain	GLY	9	5.32	6.6	0	0
Ab-Heavy Chain	GLY	10	4.59	5.7	4.59	14.2
Ab-Heavy Chain	LEU	11	41.93	23.5	15.63	11.1
Ab-Heavy Chain	VAL	12	3.42	2.3	3.42	3
Ab-Heavy Chain	GLN	13	142.65	79.9	139.44	98.9
Ab-Heavy Chain	PRO	14	55.44	40.7	41.45	34.6
Ab-Heavy Chain	GLY	15	47.61	59.4	20.13	62.3
Ab-Heavy Chain	GLY	16	18.89	23.6	18.89	58.4
Ab-Heavy Chain	SER	17	68.65	58.9	47.58	60.9
Ab-Heavy Chain	LEU	18	35.58	19.9	35.58	25.2
Ab-Heavy Chain	ARG	19	130.34	54.6	121.97	60.6
Ab-Heavy Chain	LEU	20	0	0	0	0
Ab-Heavy Chain	SER	21	18.73	16.1	18.73	24
Ab-Heavy Chain	CYS	22	0	0	0	0
Ab-Heavy Chain	ALA	23	36.81	34.1	36.81	53
Ab-Heavy Chain	ALA	24	6.64	6.2	0	0
Ab-Heavy Chain	SER	25	58.85	50.5	58.85	75.3
Ab-Heavy Chain	GLY	26	74.75	93.3	29.77	92.1

Ab-Heavy Chain	PHE	27	18.56	9.3	18.5	11.3
Ab-Heavy Chain	ASN	28	102.03	70.9	102.03	96
Ab-Heavy Chain	ILE	29	0	0	0	0
Ab-Heavy Chain	LYS	30	19.95	9.9	18.79	11.5
Ab-Heavy Chain	ASP	31	78.89	56.2	78.89	76.8
Ab-Heavy Chain	THR	32	0	0	0	0
Ab-Heavy Chain	TYR	33	9.8	4.6	9.8	5.5
Ab-Heavy Chain	ILE	34	0	0	0	0
Ab-Heavy Chain	HIS	35	0	0	0	0
Ab-Heavy Chain	TRP	36	0	0	0	0
Ab-Heavy Chain	VAL	37	0	0	0	0
Ab-Heavy Chain	ARG	38	3.32	1.4	3.32	1.6
Ab-Heavy Chain	GLN	39	13.64	7.6	13.64	9.7
Ab-Heavy Chain	ALA	40	17.51	16.2	17.51	25.2
Ab-Heavy Chain	PRO	41	82.95	60.9	70.63	58.9
Ab-Heavy Chain	GLY	42	100.27	125.2	53.98	167
Ab-Heavy Chain	LYS	43	156.07	77.7	154.79	94.8
Ab-Heavy Chain	GLY	44	14.3	17.9	5.24	16.2
Ab-Heavy Chain	LEU	45	5.92	3.3	0	0
Ab-Heavy Chain	GLU	46	61.07	35.5	61.07	45.3
Ab-Heavy Chain	TRP	47	0.72	0.3	0.02	0
Ab-Heavy Chain	VAL	48	0	0	0	0
Ab-Heavy Chain	ALA	49	0	0	0	0
Ab-Heavy Chain	ARG	50	0	0	0	0
Ab-Heavy Chain	ILE	51	0.08	0	0.08	0.1
Ab-Heavy Chain	TYR	52	33.01	15.5	33.01	18.6
Ab-Heavy Chain	PRO	53	9.48	7	0	0
Ab-Heavy Chain	THR	54	108.3	77.8	72.25	71
Ab-Heavy Chain	ASN	55	83.25	57.8	57.94	54.5
Ab-Heavy Chain	GLY	56	40.76	50.9	22.38	69.2
Ab-Heavy Chain	TYR	57	70.85	33.3	70.85	39.9
Ab-Heavy Chain	THR	58	38.67	27.8	26.15	25.7
Ab-Heavy Chain	ARG	59	29.31	12.3	29.31	14.6
Ab-Heavy Chain	TYR	60	20.24	9.5	18.22	10.3
Ab-Heavy Chain	ALA	61	6.34	5.9	6.34	9.1
Ab-Heavy Chain	ASP	62	140.21	99.9	114.55	111.5
Ab-Heavy Chain	SER	63	47.2	40.5	41.79	53.5
Ab-Heavy Chain	VAL	64	0	0	0	0
Ab-Heavy Chain	LYS	65	153.01	76.2	133.21	81.6
Ab-Heavy Chain	GLY	66	86.95	108.6	49.92	154.4
Ab-Heavy Chain	ARG	67	30.98	13	30.98	15.4
Ab-Heavy Chain	PHE	68	0.34	0.2	0	0
Ab-Heavy Chain	THR	69	66.13	47.5	66.13	65
Ab-Heavy Chain	ILE	70	1.65	0.9	0	0
Ab-Heavy Chain	SER	71	34.76	29.8	34.76	44.5

Ab-Heavy Chain	ALA	72	29.48	27.3	0	0
Ab-Heavy Chain	ASP	73	41.05	29.2	41.05	40
Ab-Heavy Chain	THR	74	81.59	58.6	73.41	72.2
Ab-Heavy Chain	SER	75	124.07	106.5	89.83	115
Ab-Heavy Chain	LYS	76	162.36	80.9	147.99	90.6
Ab-Heavy Chain	ASN	77	21.61	15	21.61	20.3
Ab-Heavy Chain	THR	78	6.54	4.7	6.54	6.4
Ab-Heavy Chain	ALA	79	0	0	0	0
Ab-Heavy Chain	TYR	80	8.6	4	8.6	4.8
Ab-Heavy Chain	LEU	81	0	0	0	0
Ab-Heavy Chain	GLN	82	46.69	26.2	46.69	33.1
Ab-Heavy Chain	MET	83	0	0	0	0
Ab-Heavy Chain	ASN	84	60.38	41.9	60.38	56.8
Ab-Heavy Chain	SER	85	34.88	29.9	34.35	44
Ab-Heavy Chain	LEU	86	0	0	0	0
Ab-Heavy Chain	ARG	87	139.79	58.5	139.79	69.5
Ab-Heavy Chain	ALA	88	82.86	76.8	72.32	104.2
Ab-Heavy Chain	GLU	89	158.8	92.2	152.08	112.9
Ab-Heavy Chain	ASP	90	0	0	0	0
Ab-Heavy Chain	THR	91	16.63	11.9	16.63	16.3
Ab-Heavy Chain	ALA	92	0	0	0	0
Ab-Heavy Chain	VAL	93	26.75	17.7	26.75	23.4
Ab-Heavy Chain	TYR	94	0	0	0	0
Ab-Heavy Chain	TYR	95	0.28	0.1	0.28	0.2
Ab-Heavy Chain	CYS	96	0	0	0	0
Ab-Heavy Chain	SER	97	0	0	0	0
Ab-Heavy Chain	ARG	98	24.56	10.3	24.56	12.2
Ab-Heavy Chain	TRP	99	0	0	0	0
Ab-Heavy Chain	GLY	100	1.22	1.5	0	0
Ab-Heavy Chain	GLY	101	20.41	25.5	18.86	58.3
Ab-Heavy Chain	ASP	102	89.76	63.9	84.08	81.9
Ab-Heavy Chain	GLY	103	0	0	0	0
Ab-Heavy Chain	PHE	104	13.8	6.9	13.8	8.4
Ab-Heavy Chain	TYR	105	4.79	2.3	4.79	2.7
Ab-Heavy Chain	ALA	106	0	0	0	0
Ab-Heavy Chain	MET	107	0	0	0	0
Ab-Heavy Chain	ASP	108	5.26	3.7	3.19	3.1
Ab-Heavy Chain	TYR	109	43.31	20.4	43.31	24.4
Ab-Heavy Chain	TRP	110	15.58	6.2	0	0
Ab-Heavy Chain	GLY	111	0.01	0	0	0
Ab-Heavy Chain	GLN	112	85.63	48	79.61	56.5
Ab-Heavy Chain	GLY	113	11.56	14.4	0	0
Ab-Heavy Chain	THR	114	8.53	6.1	8.53	8.4
Ab-Heavy Chain	LEU	115	4.95	2.8	4.31	3.1
Ab-Heavy Chain	VAL	116	0	0	0	0

Ab-Heavy Chain	THR	117	0.61	0.4	0.61	0.6
Ab-Heavy Chain	VAL	118	3.26	2.2	0	0
Ab-Heavy Chain	SER	119	24.75	21.2	24.75	31.7
Ab-Heavy Chain	SER	120	105.32	90.4	83.76	107.2
Ab-Heavy Chain	ALA	121	43.23	40	42.58	61.3
Ab-Heavy Chain	SER	122	93.81	80.5	85.88	109.9
Ab-Heavy Chain	THR	123	55.08	39.5	33.38	32.8
Ab-Heavy Chain	LYS	124	125.1	62.3	125.1	76.6
Ab-Heavy Chain	GLY	125	28.88	36.1	6.82	21.1
Ab-Heavy Chain	PRO	126	9.57	7	0	0
Ab-Heavy Chain	SER	127	43.91	37.7	43.91	56.2
Ab-Heavy Chain	VAL	128	6.05	4	0	0
Ab-Heavy Chain	PHE	129	3.69	1.8	3.69	2.2
Ab-Heavy Chain	PRO	130	14.99	11	14.99	12.5
Ab-Heavy Chain	LEU	131	0	0	0	0
Ab-Heavy Chain	ALA	132	10.64	9.9	10.64	15.3
Ab-Heavy Chain	PRO	133	0	0	0	0
Ab-Heavy Chain	SER	134	16.08	13.8	15.67	20.1
Ab-Heavy Chain	SER	135	95.15	81.7	82.04	105
Ab-Heavy Chain	LYS	136	74.47	37.1	74.47	45.6
Ab-Heavy Chain	SER	137	0	0	0	0
Ab-Heavy Chain	THR	138	35.41	25.4	21.48	21.1
Ab-Heavy Chain	SER	139	42.44	36.4	36.16	46.3
Ab-Heavy Chain	GLY	140	99.33	124	57.6	178.2
Ab-Heavy Chain	GLY	141	63.67	79.5	43.65	135
Ab-Heavy Chain	THR	142	42.68	30.6	42.68	42
Ab-Heavy Chain	ALA	143	0	0	0	0
Ab-Heavy Chain	ALA	144	0	0	0	0
Ab-Heavy Chain	LEU	145	0	0	0	0
Ab-Heavy Chain	GLY	146	0	0	0	0
Ab-Heavy Chain	CYS	147	0	0	0	0
Ab-Heavy Chain	LEU	148	0	0	0	0
Ab-Heavy Chain	VAL	149	0	0	0	0
Ab-Heavy Chain	LYS	150	5.35	2.7	5.35	3.3
Ab-Heavy Chain	ASP	151	24.9	17.7	24.9	24.3
Ab-Heavy Chain	TYR	152	0	0	0	0
Ab-Heavy Chain	PHE	153	8.93	4.5	8.93	5.4
Ab-Heavy Chain	PRO	154	0	0	0	0
Ab-Heavy Chain	GLU	155	32.93	19.1	32.93	24.4
Ab-Heavy Chain	PRO	156	42.62	31.3	42.62	35.5
Ab-Heavy Chain	VAL	157	11.03	7.3	0	0
Ab-Heavy Chain	THR	158	77.05	55.3	77.05	75.8
Ab-Heavy Chain	VAL	159	17.51	11.6	0	0
Ab-Heavy Chain	SER	160	29.27	25.1	29.27	37.5
Ab-Heavy Chain	TRP	161	0	0	0	0

Ab-Heavy Chain	ASN	162	19.37	13.5	15.7	14.8
Ab-Heavy Chain	SER	163	117	100.4	80.92	103.6
Ab-Heavy Chain	GLY	164	40.85	51	19.55	60.5
Ab-Heavy Chain	ALA	165	109.93	101.8	73.46	105.8
Ab-Heavy Chain	LEU	166	18.46	10.3	18.46	13.1
Ab-Heavy Chain	THR	167	100.48	72.1	91.17	89.6
Ab-Heavy Chain	SER	168	95.43	81.9	93.69	120
Ab-Heavy Chain	GLY	169	26.1	32.6	16.23	50.2
Ab-Heavy Chain	VAL	170	26.55	17.5	11.64	10.2
Ab-Heavy Chain	HIS	171	5.62	3.1	5.62	3.8
Ab-Heavy Chain	THR	172	47.58	34.2	28.94	28.5
Ab-Heavy Chain	PHE	173	0.01	0	0	0
Ab-Heavy Chain	PRO	174	61.25	45	61.25	51.1
Ab-Heavy Chain	ALA	175	20.28	18.8	1.6	2.3
Ab-Heavy Chain	VAL	176	17.4	11.5	17.4	15.2
Ab-Heavy Chain	LEU	177	110.84	62.1	105.72	74.9
Ab-Heavy Chain	GLN	178	5.09	2.9	5.05	3.6
Ab-Heavy Chain	SER	179	143.33	123	105.16	134.6
Ab-Heavy Chain	SER	180	85.32	73.2	49.55	63.4
Ab-Heavy Chain	GLY	181	25.93	32.4	25.93	80.2
Ab-Heavy Chain	LEU	182	18.74	10.5	18.74	13.3
Ab-Heavy Chain	TYR	183	35.13	16.5	35.13	19.8
Ab-Heavy Chain	SER	184	0	0	0	0
Ab-Heavy Chain	LEU	185	1.89	1.1	1.89	1.3
Ab-Heavy Chain	SER	186	0	0	0	0
Ab-Heavy Chain	SER	187	0	0	0	0
Ab-Heavy Chain	VAL	188	0	0	0	0
Ab-Heavy Chain	VAL	189	0	0	0	0
Ab-Heavy Chain	THR	190	21	15.1	14.85	14.6
Ab-Heavy Chain	VAL	191	0.55	0.4	0.26	0.2
Ab-Heavy Chain	PRO	192	78.58	57.7	78.58	65.5
Ab-Heavy Chain	SER	193	33.8	29	24.23	31
Ab-Heavy Chain	SER	194	117.82	101.1	97.99	125.5
Ab-Heavy Chain	SER	195	3.74	3.2	3.74	4.8
Ab-Heavy Chain	LEU	196	36.09	20.2	17.34	12.3
Ab-Heavy Chain	GLY	197	80.49	100.5	30.42	94.1
Ab-Heavy Chain	THR	198	131.69	94.6	97.95	96.3
Ab-Heavy Chain	GLN	199	95.7	53.6	95.7	67.9
Ab-Heavy Chain	THR	200	86.83	62.3	86.83	85.4
Ab-Heavy Chain	TYR	201	0.17	0.1	0.17	0.1
Ab-Heavy Chain	ILE	202	48.16	27.5	48.16	34.9
Ab-Heavy Chain	CYS	203	0	0	0	0
Ab-Heavy Chain	ASN	204	12.64	8.8	12.64	11.9
Ab-Heavy Chain	VAL	205	0	0	0	0
Ab-Heavy Chain	ASN	206	46.49	32.3	46.49	43.8

Ab-Heavy Chain	HIS	207	0	0	0	0
Ab-Heavy Chain	LYS	208	92.39	46	90.66	55.5
Ab-Heavy Chain	PRO	209	30.03	22.1	3.34	2.8
Ab-Heavy Chain	SER	210	34.13	29.3	5.69	7.3
Ab-Heavy Chain	ASN	211	123.57	85.8	104.29	98.2
Ab-Heavy Chain	THR	212	25.53	18.3	25.53	25.1
Ab-Heavy Chain	LYS	213	154.96	77.2	124	75.9
Ab-Heavy Chain	VAL	214	34.77	23	34.77	30.4
Ab-Heavy Chain	ASP	215	109.45	78	77.12	75.1
Ab-Heavy Chain	LYS	216	61.42	30.6	61.42	37.6
Ab-Heavy Chain	LYS	217	35.95	17.9	32.02	19.6
Ab-Heavy Chain	VAL	218	0	0	0	0
Ab-Heavy Chain	GLU	219	114.66	66.6	110.73	82.2
Ab-Heavy Chain	PRO	220	117.22	86.1	46.42	38.7
HER2	THR	1	110.99	79.7	58.86	57.9
HER2	GLN	2	109.27	61.2	108.51	77
HER2	VAL	3	25.17	16.6	13.49	11.8
HER2	CYS	4	2.11	1.6	2.03	2.1
HER2	THR	5	21.08	15.1	19.34	19
HER2	GLY	6	0	0	0	0
HER2	THR	7	0.35	0.3	0	0
HER2	ASP	8	51.66	36.8	50.6	49.3
HER2	MET	9	0	0	0	0
HER2	LYS	10	83.35	41.5	83.35	51
HER2	LEU	11	60.86	34.1	52.69	37.3
HER2	ARG	12	121.36	50.8	121.36	60.3
HER2	LEU	13	140.19	78.5	124.64	88.3
HER2	PRO	14	0	0	0	0
HER2	ALA	15	38.92	36.1	18.73	27
HER2	SER	16	0.21	0.2	0.21	0.3
HER2	PRO	17	65.12	47.8	65.07	54.3
HER2	GLU	18	107.98	62.7	106.07	78.7
HER2	THR	19	0.21	0.2	0.21	0.2
HER2	HIS	20	0	0	0	0
HER2	LEU	21	24.31	13.6	24.31	17.2
HER2	ASP	22	51.71	36.8	51.71	50.4
HER2	MET	23	0	0	0	0
HER2	LEU	24	0	0	0	0
HER2	ARG	25	96.28	40.3	96.28	47.8
HER2	HIS	26	40.07	21.9	39.85	27.1
HER2	LEU	27	1	0.6	1	0.7
HER2	TYR	28	0	0	0	0
HER2	GLN	29	113.61	63.6	103.43	73.4
HER2	GLY	30	33.66	42	27.21	84.2
HER2	CYS	31	0	0	0	0

HER2	GLN	32	46.13	25.8	46.13	32.7
HER2	VAL	33	6.81	4.5	6.81	6
HER2	VAL	34	0	0	0	0
HER2	GLN	35	3.82	2.1	3.44	2.4
HER2	GLY	36	2.98	3.7	2.98	9.2
HER2	ASN	37	0	0	0	0
HER2	LEU	38	0	0	0	0
HER2	GLU	39	0	0	0	0
HER2	LEU	40	0	0	0	0
HER2	THR	41	0	0	0	0
HER2	TYR	42	40.42	19	37.94	21.4
HER2	LEU	43	0	0	0	0
HER2	PRO	44	23.09	17	23.09	19.3
HER2	THR	45	54.79	39.3	48.44	47.6
HER2	ASN	46	144.47	100.4	119.82	112.8
HER2	ALA	47	11.34	10.5	0	0
HER2	SER	48	86.57	74.3	86.57	110.8
HER2	LEU	49	2.85	1.6	0	0
HER2	SER	50	60.57	52	60.26	77.1
HER2	PHE	51	0.24	0.1	0.24	0.1
HER2	LEU	52	0	0	0	0
HER2	GLN	53	89.17	50	89.17	63.2
HER2	ASP	54	40.67	29	40.67	39.6
HER2	ILE	55	0	0	0	0
HER2	GLN	56	57.2	32	57.2	40.6
HER2	GLU	57	3.6	2.1	3.6	2.7
HER2	VAL	58	0	0	0	0
HER2	GLN	59	31.54	17.7	31.54	22.4
HER2	GLY	60	0	0	0	0
HER2	TYR	61	0.32	0.1	0.32	0.2
HER2	VAL	62	0	0	0	0
HER2	LEU	63	0	0	0	0
HER2	ILE	64	0	0	0	0
HER2	ALA	65	0	0	0	0
HER2	HIS	66	57.11	31.2	55.52	37.7
HER2	ASN	67	0	0	0	0
HER2	GLN	68	41.68	23.3	41.68	29.6
HER2	VAL	69	1.17	0.8	1.17	1
HER2	ARG	70	65.3	27.3	65.22	32.4
HER2	GLN	71	94.44	52.9	93.99	66.7
HER2	VAL	72	0	0	0	0
HER2	PRO	73	11.94	8.8	11.94	10
HER2	LEU	74	0	0	0	0
HER2	GLN	75	28.23	15.8	27.56	19.5
HER2	ARG	76	146.71	61.4	146.2	72.6

HER2	LEU	77	0	0	0	0
HER2	ARG	78	36.06	15.1	36.06	17.9
HER2	ILE	79	0	0	0	0
HER2	VAL	80	0	0	0	0
HER2	ARG	81	6.13	2.6	6.13	3
HER2	GLY	82	0.01	0	0	0
HER2	THR	83	77.73	55.8	43.62	42.9
HER2	GLN	84	68.95	38.6	68.95	48.9
HER2	LEU	85	34.43	19.3	27.99	19.8
HER2	PHE	86	1.59	0.8	1.59	1
HER2	GLU	87	107.13	62.2	100.58	74.6
HER2	ASP	88	143.39	102.1	127.55	124.2
HER2	ASN	89	60.16	41.8	59.09	55.6
HER2	TYR	90	8.94	4.2	8.94	5
HER2	ALA	91	0	0	0	0
HER2	LEU	92	0	0	0	0
HER2	ALA	93	0	0	0	0
HER2	VAL	94	0	0	0	0
HER2	LEU	95	0	0	0	0
HER2	ASP	96	29.75	21.2	29.75	29
HER2	ASN	97	0	0	0	0
HER2	GLY	98	4.2	5.2	0.42	1.3
HER2	ASP	99	77.96	55.5	54.93	53.5
HER2	PRO	100	104.76	77	89.12	74.3
HER2	LEU	101	301.94	169	230.73	163.5
HER2	SER	111	165.38	142	109.73	140.5
HER2	PRO	112	66.84	49.1	66.84	55.7
HER2	GLY	113	2.2	2.8	0.16	0.5
HER2	GLY	114	0	0	0	0
HER2	LEU	115	0	0	0	0
HER2	ARG	116	74.76	31.3	74.76	37.1
HER2	GLU	117	6.05	3.5	6.05	4.5
HER2	LEU	118	0	0	0	0
HER2	GLN	119	15.19	8.5	15.19	10.8
HER2	LEU	120	0	0	0	0
HER2	ARG	121	70.74	29.6	66	32.8
HER2	SER	122	12.18	10.5	12.18	15.6
HER2	LEU	123	0	0	0	0
HER2	THR	124	0	0	0	0
HER2	GLU	125	0	0	0	0
HER2	ILE	126	0	0	0	0
HER2	LEU	127	7.29	4.1	7.29	5.2
HER2	LYS	128	131.34	65.4	131.34	80.4
HER2	GLY	129	7.66	9.6	3.93	12.1
HER2	GLY	130	0	0	0	0

HER2	VAL	131	0	0	0	0
HER2	LEU	132	5.85	3.3	5.85	4.1
HER2	ILE	133	0	0	0	0
HER2	GLN	134	21.53	12.1	21.53	15.3
HER2	ARG	135	150.08	62.9	150.08	74.6
HER2	ASN	136	0	0	0	0
HER2	PRO	137	32.15	23.6	32.15	26.8
HER2	GLN	138	1.42	0.8	1.42	1
HER2	LEU	139	0	0	0	0
HER2	CYS	140	0	0	0	0
HER2	TYR	141	1.38	0.6	1.26	0.7
HER2	GLN	142	0	0	0	0
HER2	ASP	143	90.53	64.5	59.39	57.8
HER2	THR	144	7.48	5.4	6.54	6.4
HER2	ILE	145	5.56	3.2	0	0
HER2	LEU	146	31.88	17.8	31.88	22.6
HER2	TRP	147	12.54	5	12	5.7
HER2	LYS	148	129.17	64.3	127.26	77.9
HER2	ASP	149	0	0	0	0
HER2	ILE	150	0	0	0	0
HER2	PHE	151	1.7	0.9	1.7	1
HER2	HIS	152	7.88	4.3	7.88	5.4
HER2	LYS	153	125.6	62.5	114.86	70.3
HER2	ASN	154	131.28	91.2	109.29	102.9
HER2	ASN	155	5.85	4.1	5.85	5.5
HER2	GLN	156	55.19	30.9	38.06	27
HER2	LEU	157	217.24	121.6	194.83	138.1
HER2	ALA	158	15.34	14.2	5.23	7.5
HER2	LEU	159	89.39	50	88.62	62.8
HER2	THR	160	56.75	40.8	28.71	28.2
HER2	LEU	161	95.47	53.4	95.47	67.7
HER2	ILE	162	34.37	19.6	6.39	4.6
HER2	ASP	163	35.25	25.1	35.25	34.3
HER2	THR	164	56.2	40.4	47.51	46.7
HER2	ASN	165	132.18	91.8	128.89	121.3
HER2	ARG	166	41.31	17.3	33.27	16.5
HER2	SER	167	105.94	90.9	66.2	84.8
HER2	ARG	168	32.74	13.7	31.18	15.5
HER2	ALA	169	107.95	100	96.25	138.7
HER2	CYS	170	37.52	27.9	5.45	5.6
HER2	HIS	171	143.22	78.3	142.47	96.9
HER2	PRO	172	128.72	94.6	125	104.3
HER2	CYS	173	3.09	2.3	0.26	0.3
HER2	SER	174	10.45	9	10.39	13.3
HER2	PRO	175	171.72	126.1	141.88	118.3

HER2	MET	176	167.82	86.4	146.06	93.2
HER2	CYS	177	1.78	1.3	0	0
HER2	LYS	178	216.59	107.9	188.66	115.5
HER2	GLY	179	26.85	33.5	18.79	58.1
HER2	SER	180	81.51	70	76.7	98.2
HER2	ARG	181	82.37	34.5	82.37	40.9
HER2	CYS	182	0	0	0	0
HER2	TRP	183	0	0	0	0
HER2	GLY	184	0	0	0	0
HER2	GLU	185	108.49	63	101.62	75.4
HER2	SER	186	42.49	36.5	42.49	54.4
HER2	SER	187	54.09	46.4	41.89	53.6
HER2	GLU	188	136.98	79.5	128.73	95.5
HER2	ASP	189	0	0	0	0
HER2	CYS	190	19.09	14.2	15.75	16.3
HER2	GLN	191	0.93	0.5	0	0
HER2	SER	192	63.73	54.7	63.73	81.6
HER2	LEU	193	11.63	6.5	5.42	3.8
HER2	THR	194	1	0.7	1	1
HER2	ARG	195	28.93	12.1	22.5	11.2
HER2	THR	196	62.74	45	23.84	23.4
HER2	VAL	197	47.58	31.4	44.23	38.7
HER2	CYS	198	23.85	17.8	0	0
HER2	ALA	199	21.66	20.1	17.11	24.7
HER2	GLY	200	95.26	118.9	58.2	180
HER2	GLY	201	109.84	137.1	48.5	150
HER2	CYS	202	14.08	10.5	10.14	10.5
HER2	ALA	203	10.92	10.1	10.32	14.9
HER2	ARG	204	0	0	0	0
HER2	CYS	205	0.07	0.1	0	0
HER2	LYS	206	106.37	53	71.78	44
HER2	GLY	207	7.66	9.6	7.66	23.7
HER2	PRO	208	110.64	81.3	100.5	83.8
HER2	LEU	209	124.51	69.7	124.51	88.2
HER2	PRO	210	104.01	76.4	95.14	79.4
HER2	THR	211	102.59	73.7	102.59	100.9
HER2	ASP	212	15.08	10.7	15.08	14.7
HER2	CYS	213	19.52	14.5	18.44	19.1
HER2	CYS	214	5.58	4.2	0	0
HER2	HIS	215	58.37	31.9	58.37	39.7
HER2	GLU	216	142.74	82.9	141.04	104.7
HER2	GLN	217	7.83	4.4	7.83	5.6
HER2	CYS	218	0	0	0	0
HER2	ALA	219	0	0	0	0
HER2	ALA	220	0	0	0	0

HER2	GLY	221	0	0	0	0
HER2	CYS	222	1.83	1.4	0	0
HER2	THR	223	107.4	77.1	83.22	81.8
HER2	GLY	224	2.99	3.7	2.99	9.2
HER2	PRO	225	86.98	63.9	77.82	64.9
HER2	LYS	226	140.17	69.8	140.17	85.8
HER2	HIS	227	71.38	39	71.38	48.5
HER2	SER	228	55.72	47.8	29.73	38.1
HER2	ASP	229	29.75	21.2	29.64	28.9
HER2	CYS	230	23.93	17.8	0	0
HER2	LEU	231	42.63	23.9	36.74	26
HER2	ALA	232	2.07	1.9	2.07	3
HER2	CYS	233	0.6	0.4	0.6	0.6
HER2	LEU	234	18.49	10.3	17.34	12.3
HER2	HIS	235	65.69	35.9	65.02	44.2
HER2	PHE	236	43.79	22	43.79	26.7
HER2	ASN	237	42.51	29.5	42.06	39.6
HER2	HIS	238	21.48	11.7	21.48	14.6
HER2	SER	239	100.56	86.3	77.65	99.4
HER2	GLY	240	46.11	57.6	35.23	109
HER2	ILE	241	60.97	34.8	60.97	44.2
HER2	CYS	242	0.84	0.6	0.84	0.9
HER2	GLU	243	50.2	29.1	50.2	37.3
HER2	LEU	244	114.05	63.8	111.35	78.9
HER2	HIS	245	130.96	71.6	130.77	88.9
HER2	CYS	246	3.97	3	0.15	0.2
HER2	PRO	247	14.37	10.6	14.37	12
HER2	ALA	248	49.32	45.7	47.27	68.1
HER2	LEU	249	26.2	14.7	22.77	16.1
HER2	VAL	250	18.2	12	17.23	15.1
HER2	THR	251	78.25	56.2	78.25	76.9
HER2	TYR	252	120.8	56.8	117.12	66
HER2	ASN	253	35.19	24.4	35.19	33.1
HER2	THR	254	143.62	103.1	128.41	126.3
HER2	ASP	255	163.02	116.1	131.33	127.9
HER2	THR	256	104.03	74.7	81.62	80.3
HER2	PHE	257	150.31	75.4	137.02	83.5
HER2	GLU	258	117.55	68.2	117.55	87.2
HER2	SER	259	87.56	75.2	62.99	80.6
HER2	MET	260	93.6	48.2	93.6	59.8
HER2	PRO	261	126.94	93.2	119.46	99.6
HER2	ASN	262	23.2	16.1	15.44	14.5
HER2	PRO	263	137.55	101	121.2	101.1
HER2	GLU	264	135.9	78.9	135.9	100.9
HER2	GLY	265	1.55	1.9	0	0

HER2	ARG	266	45.79	19.2	45.61	22.7
HER2	TYR	267	21.63	10.2	21.63	12.2
HER2	THR	268	20.7	14.9	20.7	20.4
HER2	PHE	269	2.3	1.2	2.3	1.4
HER2	GLY	270	13.82	17.2	2.48	7.7
HER2	ALA	271	2.17	2	2.17	3.1
HER2	SER	272	4.97	4.3	4.97	6.4
HER2	CYS	273	11.14	8.3	0	0
HER2	VAL	274	31.57	20.8	31.57	27.6
HER2	THR	275	109.11	78.3	96.82	95.2
HER2	ALA	276	69.38	64.3	62.64	90.2
HER2	CYS	277	5.77	4.3	0	0
HER2	PRO	278	38.84	28.5	38.84	32.4
HER2	TYR	279	87.68	41.2	78.37	44.2
HER2	ASN	280	1.09	0.8	0.49	0.5
HER2	TYR	281	11.46	5.4	11.46	6.5
HER2	LEU	282	0	0	0	0
HER2	SER	283	3.28	2.8	3.28	4.2
HER2	THR	284	0	0	0	0
HER2	ASP	285	71.94	51.2	64	62.3
HER2	VAL	286	126.83	83.8	108.06	94.6
HER2	GLY	287	0.14	0.2	0.01	0
HER2	SER	288	13.64	11.7	13.64	17.5
HER2	CYS	289	5.18	3.9	0	0
HER2	THR	290	49.11	35.3	49.11	48.3
HER2	LEU	291	35.76	20	35.23	25
HER2	VAL	292	65.7	43.4	62.74	54.9
HER2	CYS	293	16.03	11.9	3.01	3.1
HER2	PRO	294	45.28	33.3	44.42	37
HER2	LEU	295	178	99.6	169.74	120.3
HER2	HIS	296	135.36	74	129.99	88.4
HER2	ASN	297	13.79	9.6	2.26	2.1
HER2	GLN	298	38.08	21.3	38.08	27
HER2	GLU	299	15.25	8.9	0	0
HER2	VAL	300	38.99	25.7	38.99	34.1
HER2	THR	301	111.23	79.9	77.2	75.9
HER2	ALA	302	98.1	90.9	29.71	42.8
HER2	THR	306	107.23	77	75.49	74.2
HER2	GLN	307	1.83	1	1.83	1.3
HER2	ARG	308	105.78	44.3	105.78	52.6
HER2	CYS	309	0	0	0	0
HER2	GLU	310	63.81	37	63.81	47.4
HER2	LYS	311	141.69	70.6	137.68	84.3
HER2	CYS	312	11.8	8.8	0	0
HER2	SER	313	133.79	114.8	95.79	122.6

HER2	LYS	314	201.73	100.5	201.5	123.4
HER2	PRO	315	143.89	105.7	138.25	115.3
HER2	CYS	316	45.1	33.6	31.8	32.9
HER2	ALA	317	69.5	64.4	68.44	98.6
HER2	ARG	318	292.25	122.4	262.93	130.6
HER2	VAL	319	42	27.7	25.34	22.2
HER2	CYS	320	10.53	7.8	10.27	10.6
HER2	TYR	321	47.65	22.4	47.46	26.8
HER2	GLY	322	0	0	0	0
HER2	LEU	323	0	0	0	0
HER2	GLY	324	29.62	37	11.68	36.1
HER2	MET	325	13.01	6.7	13.01	8.3
HER2	GLU	326	93.67	54.4	92.55	68.7
HER2	HIS	327	124.84	68.3	106.27	72.3
HER2	LEU	328	3.55	2	3.55	2.5
HER2	ARG	329	192.39	80.6	173.93	86.4
HER2	GLU	330	198.37	115.2	177.71	131.9
HER2	VAL	331	47.08	31.1	42.13	36.9
HER2	ARG	332	186.83	78.2	182	90.4
HER2	ALA	333	0	0	0	0
HER2	VAL	334	0	0	0	0
HER2	THR	335	12.47	9	12.47	12.3
HER2	SER	336	23.7	20.3	5.56	7.1
HER2	ALA	337	93.03	86.2	66.45	95.7
HER2	ASN	338	5.66	3.9	5.66	5.3
HER2	ILE	339	0	0	0	0
HER2	GLN	340	118.42	66.3	108.96	77.3
HER2	GLU	341	71.99	41.8	59.35	44
HER2	PHE	342	0	0	0	0
HER2	ALA	343	70.64	65.4	47.57	68.5
HER2	GLY	344	60.22	75.2	35.21	108.9
HER2	CYS	345	15.98	11.9	15.98	16.5
HER2	LYS	346	152.91	76.1	149.09	91.3
HER2	LYS	347	41.64	20.7	40.15	24.6
HER2	ILE	348	0	0	0	0
HER2	PHE	349	6.27	3.1	6.27	3.8
HER2	GLY	350	0	0	0	0
HER2	SER	351	0	0	0	0
HER2	LEU	352	0	0	0	0
HER2	ALA	353	0	0	0	0
HER2	PHE	354	0	0	0	0
HER2	LEU	355	11.33	6.3	11.33	8
HER2	PRO	356	78.57	57.7	78.57	65.5
HER2	GLU	357	31.5	18.3	31.5	23.4
HER2	SER	358	0	0	0	0

HER2	PHE	359	4.34	2.2	4.18	2.5
HER2	ASP	360	181.82	129.5	130.55	127.1
HER2	SER	365	123.11	105.7	28.71	36.8
HER2	ASN	366	208.67	145	172.24	162.1
HER2	THR	367	60.81	43.7	60.69	59.7
HER2	ALA	368	72.2	66.9	68.87	99.2
HER2	PRO	369	47.29	34.7	46.87	39.1
HER2	LEU	370	1.09	0.6	0	0
HER2	GLN	371	106.01	59.4	106.01	75.2
HER2	PRO	372	23.4	17.2	23.4	19.5
HER2	GLU	373	126.84	73.6	119.59	88.8
HER2	GLN	374	41.19	23.1	41.17	29.2
HER2	LEU	375	0	0	0	0
HER2	GLN	376	110.33	61.8	109.93	78
HER2	VAL	377	14.44	9.5	14.18	12.4
HER2	PHE	378	0	0	0	0
HER2	GLU	379	115.03	66.8	115.03	85.4
HER2	THR	380	48.87	35.1	48.87	48.1
HER2	LEU	381	0	0	0	0
HER2	GLU	382	57.31	33.3	57.31	42.5
HER2	GLU	383	13.99	8.1	13.99	10.4
HER2	ILE	384	0	0	0	0
HER2	THR	385	0	0	0	0
HER2	GLY	386	0	0	0	0
HER2	TYR	387	18.19	8.6	18.19	10.3
HER2	LEU	388	0	0	0	0
HER2	TYR	389	21.86	10.3	21.86	12.3
HER2	ILE	390	0	0	0	0
HER2	SER	391	13.22	11.3	4.25	5.4
HER2	ALA	392	12	11.1	12	17.3
HER2	TRP	393	3.98	1.6	0	0
HER2	PRO	394	8.48	6.2	8.48	7.1
HER2	ASP	395	145.47	103.6	125.35	122.1
HER2	SER	396	101.53	87.1	77.85	99.7
HER2	LEU	397	25.08	14	25.08	17.8
HER2	PRO	398	53.2	39.1	53.2	44.4
HER2	ASP	399	3.71	2.6	3.71	3.6
HER2	LEU	400	0	0	0	0
HER2	SER	401	30.7	26.4	29.24	37.4
HER2	VAL	402	3.7	2.4	3.7	3.2
HER2	PHE	403	0	0	0	0
HER2	GLN	404	44.38	24.9	44.38	31.5
HER2	ASN	405	42.99	29.9	42.99	40.5
HER2	LEU	406	0	0	0	0
HER2	GLN	407	64.49	36.1	64.49	45.7

HER2	VAL	408	1.12	0.7	1.12	1
HER2	ILE	409	0	0	0	0
HER2	ARG	410	11.04	4.6	11.04	5.5
HER2	GLY	411	0.1	0.1	0	0
HER2	ARG	412	6.78	2.8	0	0
HER2	ILE	413	37.95	21.7	37.95	27.5
HER2	LEU	414	5.35	3	1.07	0.8
HER2	HIS	415	16.81	9.2	16.81	11.4
HER2	ASN	416	3.38	2.3	0.98	0.9
HER2	GLY	417	3.53	4.4	3.53	10.9
HER2	ALA	418	0	0	0	0
HER2	TYR	419	0	0	0	0
HER2	SER	420	0	0	0	0
HER2	LEU	421	0	0	0	0
HER2	THR	422	0	0	0	0
HER2	LEU	423	0	0	0	0
HER2	GLN	424	18.28	10.2	18.24	12.9
HER2	GLY	425	52.36	65.4	23.45	72.5
HER2	LEU	426	0.29	0.2	0	0
HER2	GLY	427	10.56	13.2	4.5	13.9
HER2	ILE	428	0	0	0	0
HER2	SER	429	30.97	26.6	30.97	39.6
HER2	TRP	430	55.6	22.3	55.6	26.3
HER2	LEU	431	0	0	0	0
HER2	GLY	432	0	0	0	0
HER2	LEU	433	0	0	0	0
HER2	ARG	434	25.04	10.5	15.6	7.8
HER2	SER	435	25.34	21.8	21.84	28
HER2	LEU	436	0.12	0.1	0	0
HER2	ARG	437	123.83	51.9	123.83	61.5
HER2	GLU	438	1.23	0.7	1.23	0.9
HER2	LEU	439	0	0	0	0
HER2	GLY	440	0	0	0	0
HER2	SER	441	1.84	1.6	1.84	2.4
HER2	GLY	442	1.39	1.7	0	0
HER2	LEU	443	0	0	0	0
HER2	ALA	444	0	0	0	0
HER2	LEU	445	0	0	0	0
HER2	ILE	446	0	0	0	0
HER2	HIS	447	2.41	1.3	2.41	1.6
HER2	HIS	448	86.79	47.5	84.87	57.7
HER2	ASN	449	0	0	0	0
HER2	THR	450	100.03	71.8	99.9	98.2
HER2	HIS	451	99.84	54.6	99.84	67.9
HER2	LEU	452	0	0	0	0

HER2	CYS	453	0	0	0	0
HER2	PHE	454	0.14	0.1	0	0
HER2	VAL	455	0	0	0	0
HER2	HIS	456	71.62	39.2	39.15	26.6
HER2	THR	457	9.08	6.5	9.08	8.9
HER2	VAL	458	2.84	1.9	0	0
HER2	PRO	459	5.16	3.8	5.16	4.3
HER2	TRP	460	14.68	5.9	13.16	6.2
HER2	ASP	461	152.24	108.4	140.04	136.4
HER2	GLN	462	86.78	48.6	84.08	59.6
HER2	LEU	463	0	0	0	0
HER2	PHE	464	16.94	8.5	9.68	5.9
HER2	ARG	465	92.96	38.9	67.85	33.7
HER2	ASN	466	33.54	23.3	29.68	27.9
HER2	PRO	467	116.73	85.8	99.04	82.6
HER2	HIS	468	1.89	1	1.89	1.3
HER2	GLN	469	8.88	5	0	0
HER2	ALA	470	15.69	14.5	15.69	22.6
HER2	LEU	471	18.33	10.3	5.71	4
HER2	LEU	472	3.26	1.8	3.26	2.3
HER2	HIS	473	92.4	50.5	81.36	55.3
HER2	THR	474	32.51	23.3	32.51	32
HER2	ALA	475	51.64	47.8	40.95	59
HER2	ASN	476	4.67	3.2	0.17	0.2
HER2	ARG	477	42.28	17.7	42.23	21
HER2	PRO	478	79.9	58.7	79.9	66.6
HER2	GLU	479	113.67	66	109.72	81.4
HER2	ASP	480	116.86	83.2	114.37	111.4
HER2	GLU	481	94.94	55.1	94.94	70.5
HER2	CYS	482	0.67	0.5	0.67	0.7
HER2	VAL	483	96.15	63.5	86.97	76.1
HER2	GLY	484	86.05	107.4	34.26	106
HER2	GLU	485	130.47	75.7	103.18	76.6
HER2	GLY	486	64.37	80.4	31.56	97.6
HER2	LEU	487	31.81	17.8	30.06	21.3
HER2	ALA	488	59.59	55.2	54.59	78.7
HER2	CYS	489	12.22	9.1	0.62	0.6
HER2	HIS	490	65.17	35.6	64.06	43.6
HER2	GLN	491	192.74	108	176.7	125.3
HER2	LEU	492	15.6	8.7	15.6	11.1
HER2	CYS	493	2.84	2.1	0	0
HER2	ALA	494	16.69	15.5	16.69	24
HER2	ARG	495	263.03	110.2	234.92	116.7
HER2	GLY	496	48.72	60.8	31.24	96.6
HER2	HIS	497	43.94	24	43.94	29.9

HER2	CYS	498	0	0	0	0
HER2	TRP	499	0	0	0	0
HER2	GLY	500	0	0	0	0
HER2	PRO	501	35.48	26.1	30.83	25.7
HER2	GLY	502	17.03	21.3	14.39	44.5
HER2	PRO	503	37.58	27.6	37.58	31.3
HER2	THR	504	48.56	34.9	48.16	47.4
HER2	GLN	505	7.55	4.2	7.55	5.4
HER2	CYS	506	2.62	1.9	0	0
HER2	VAL	507	21.69	14.3	3.84	3.4
HER2	ASN	508	97.93	68	97.93	92.2
HER2	CYS	509	0.67	0.5	0.09	0.1
HER2	SER	510	38.81	33.3	18.67	23.9
HER2	GLN	511	61.61	34.5	61.61	43.7
HER2	PHE	512	46.9	23.5	46.9	28.6
HER2	LEU	513	28.48	15.9	28.48	20.2
HER2	ARG	514	53.19	22.3	51.1	25.4
HER2	GLY	515	49.54	61.8	47.63	147.3
HER2	GLN	516	100.41	56.3	96	68.1
HER2	GLU	517	84.16	48.9	84.16	62.5
HER2	CYS	518	0	0	0	0
HER2	VAL	519	7.85	5.2	7.85	6.9
HER2	GLU	520	106.61	61.9	105.39	78.2
HER2	GLU	521	101.53	58.9	95.75	71.1
HER2	CYS	522	0.19	0.1	0	0
HER2	ARG	523	70.83	29.7	70.83	35.2
HER2	VAL	524	21.4	14.1	21.4	18.7
HER2	LEU	525	93.87	52.6	92.35	65.4
HER2	GLN	526	151.8	85	151.8	107.7
HER2	GLY	527	29.7	37.1	22.96	71
HER2	LEU	528	215.04	120.4	195.9	138.8
HER2	PRO	529	74.6	54.8	74.6	62.2
HER2	ARG	530	23.54	9.9	21.69	10.8
HER2	GLU	531	0.81	0.5	0.81	0.6
HER2	TYR	532	4.13	1.9	4.13	2.3
HER2	VAL	533	57.68	38.1	31.76	27.8
HER2	ASN	534	94.29	65.5	94.29	88.8
HER2	ALA	535	95	88	82.05	118.2
HER2	ARG	536	160.85	67.4	160.44	79.7
HER2	HIS	537	73.33	40.1	73.33	49.9
HER2	CYS	538	0.86	0.6	0	0
HER2	LEU	539	55.35	31	55.35	39.2
HER2	PRO	540	73.55	54	73.09	61
HER2	CYS	541	0.1	0.1	0.1	0.1
HER2	HIS	542	55.52	30.4	55.52	37.7

HER2	PRO	543	121.15	89	107.26	89.5
HER2	GLU	544	15.61	9.1	15.61	11.6
HER2	CYS	545	1.4	1	0	0
HER2	GLN	546	88.16	49.4	88.16	62.5
HER2	PRO	547	63.84	46.9	57.91	48.3
HER2	GLN	548	23.22	13	23.22	16.5
HER2	ASN	549	177.06	123	148.33	139.6
HER2	GLY	550	92.37	115.3	44.92	139
HER2	SER	551	54.77	47	54.77	70.1
HER2	VAL	552	53.55	35.4	52.05	45.5
HER2	THR	553	0	0	0	0
HER2	CYS	554	1.04	0.8	0	0
HER2	PHE	555	152.53	76.5	127.02	77.4
HER2	GLY	556	0.46	0.6	0.46	1.4
HER2	PRO	557	13.3	9.8	10.69	8.9
HER2	GLU	558	1.44	0.8	1.44	1.1
HER2	ALA	559	1.93	1.8	1.55	2.2
HER2	ASP	560	6.37	4.5	0	0
HER2	GLN	561	11.62	6.5	11.62	8.2
HER2	CYS	562	9.83	7.3	0	0
HER2	VAL	563	58.97	38.9	31.16	27.3
HER2	ALA	564	27.36	25.3	27.36	39.4
HER2	CYS	565	16.43	12.2	5.26	5.4
HER2	ALA	566	30.16	27.9	7.36	10.6
HER2	HIS	567	120.57	65.9	103.8	70.6
HER2	TYR	568	85.95	40.4	85.95	48.5
HER2	LYS	569	22.59	11.3	22.59	13.8
HER2	ASP	570	0	0	0	0
HER2	PRO	571	0	0	0	0
HER2	PRO	572	0.74	0.5	0	0
HER2	PHE	573	2.3	1.2	2.3	1.4
HER2	CYS	574	0	0	0	0
HER2	VAL	575	24.65	16.3	24.65	21.6
HER2	ALA	576	51.99	48.2	48.28	69.6
HER2	ARG	577	235.13	98.5	230.11	114.3
HER2	CYS	578	5.59	4.2	3.83	4
HER2	PRO	579	25.24	18.5	25.24	21.1
HER2	SER	580	112.06	96.2	49.36	63.2
HER2	ILE	591	121.09	69.1	85.09	61.7
HER2	TRP	592	150.88	60.5	149.26	70.7
HER2	LYS	593	3.19	1.6	0	0
HER2	PHE	594	88.47	44.3	88.47	53.9
HER2	PRO	595	62.93	46.2	52.26	43.6
HER2	ASP	596	26.74	19	22.22	21.6
HER2	GLU	597	220.6	128.1	190.77	141.6

HER2	GLU	598	133.32	77.4	96.17	71.4
HER2	GLY	599	5.23	6.5	5.23	16.2
HER2	ALA	600	0	0	0	0
HER2	CYS	601	0	0	0	0
HER2	GLN	602	15.48	8.7	15.48	11
HER2	PRO	603	1.24	0.9	1.24	1
HER2	CYS	604	37.59	28	32.93	34
HER2	PRO	605	113.63	83.5	88.43	73.8
HER2	ILE	606	186.31	106.4	183.4	132.9
HER2	ASN	607	215.09	149.4	166.32	156.6
